# In humans, striato-pallido-thalamic projections are largely segregated by their origin in either the striosome-like or matrix-like compartments

**DOI:** 10.3389/fnins.2023.1178473

**Published:** 2023-10-25

**Authors:** Adrian T. Funk, Asim A. O. Hassan, Norbert Brüggemann, Nutan Sharma, Hans C. Breiter, Anne J. Blood, Jeff L. Waugh

**Affiliations:** ^1^Division of Pediatric Neurology, Department of Pediatrics, University of Texas Southwestern, Dallas, TX, United States; ^2^Department of Natural Sciences and Mathematics, University of Texas at Dallas, Richardson, TX, United States; ^3^Department of Neurology and Institute of Neurogenetics, University of Lübeck, Lübeck, Germany; ^4^Department of Neurology, Massachusetts General Hospital, Harvard University, Boston, MA, United States; ^5^Laboratory of Neuroimaging and Genetics, Massachusetts General Hospital, Charlestown, MA, United States; ^6^Warren Wright Adolescent Center, Department of Psychiatry and Behavioral Sciences, Northwestern University Feinberg School of Medicine, Chicago, IL, United States; ^7^Department of Psychiatry, Massachusetts General Hospital, Harvard University, Boston, MA, United States; ^8^Mood and Motor Control Laboratory, Massachusetts General Hospital, Charlestown, MA, United States; ^9^Martinos Center for Biomedical Imaging, Massachusetts General Hospital, Charlestown, MA, United States

**Keywords:** striatum, thalamus, striosome and matrix compartments, patch, classification targets tractography, probabilistic diffusion tractography, cortico-striato-thalamo-cortical circuit, globus pallidus interna

## Abstract

Cortico-striato-thalamo-cortical (CSTC) loops are fundamental organizing units in mammalian brains. CSTCs process limbic, associative, and sensorimotor information in largely separated but interacting networks. CTSC loops pass through paired striatal compartments, striosome (aka patch) and matrix, segregated pools of medium spiny projection neurons with distinct embryologic origins, cortical/subcortical structural connectivity, susceptibility to injury, and roles in behaviors and diseases. Similarly, striatal dopamine modulates activity in striosome and matrix in opposite directions. Routing CSTCs through one compartment may be an anatomical basis for regulating discrete functions. We used differential structural connectivity, identified through probabilistic diffusion tractography, to distinguish the striatal compartments (striosome-like and matrix-like voxels) in living humans. We then mapped compartment-specific projections and quantified structural connectivity between each striatal compartment, the globus pallidus interna (GPi), and 20 thalamic nuclei in 221 healthy adults. We found that striosome-originating and matrix-originating streamlines were segregated within the GPi: striosome-like connectivity was significantly more rostral, ventral, and medial. Striato-pallido-thalamic streamline bundles that were seeded from striosome-like and matrix-like voxels transited spatially distinct portions of the white matter. Matrix-like streamlines were 5.7-fold more likely to reach the GPi, replicating animal tract-tracing studies. Striosome-like connectivity dominated in six thalamic nuclei (anteroventral, central lateral, laterodorsal, lateral posterior, mediodorsal-medial, and medial geniculate). Matrix-like connectivity dominated in seven thalamic nuclei (centromedian, parafascicular, pulvinar-anterior, pulvinar-lateral, ventral lateral-anterior, ventral lateral-posterior, ventral posterolateral). Though we mapped all thalamic nuclei independently, functionally-related nuclei were matched for compartment-level bias. We validated these results with prior thalamostriate tract tracing studies in non-human primates and other species; where reliable data was available, all agreed with our measures of structural connectivity. Matrix-like connectivity was lateralized (left > right hemisphere) in 18 thalamic nuclei, independent of handedness, diffusion protocol, sex, or whether the nucleus was striosome-dominated or matrix-dominated. Compartment-specific biases in striato-pallido-thalamic structural connectivity suggest that routing CSTC loops through striosome-like or matrix-like voxels is a fundamental mechanism for organizing and regulating brain networks. Our MRI-based assessments of striato-thalamic connectivity in humans match and extend the results of prior tract tracing studies in animals. Compartment-level characterization may improve localization of human neuropathologies and improve neurosurgical targeting in the GPi and thalamus.

## 1. Introduction

The mammalian thalamus projects to every cortical area, and each cortical area projects through intermediate structures to specific thalamic nuclei (Jones, 1998; Dufour et al., 2003). However, the thalamus is much more than a passive relay for cortical information; the thalamus integrates information from diverse cortical inputs to stabilize and reshape cortical networks (Hwang et al., 2017) and mediates direct information transfer between cortical areas (Sherman and Guillery, 2013). Precise characterization of connectivity with the thalamus is therefore essential to understanding the organization and functions of higher-order central nervous systems.

Delineating distinct thalamic nuclei has been accomplished based on histologic features (Krauth et al., 2010), structural connectivity (Behrens et al., 2003), resting state functional connectivity (Jones, 1998), and combinations of these methods (Iglesias et al., 2018; Iglehart et al., 2020). These methods have defined between 10 and 26 anatomically and functionally distinct thalamic nuclei (Iglesias et al., 2018; Ilinsky et al., 2018; Mai and Majtanik, 2018). The complexity of thalamic nuclear organization underpins the multimodal functions of cortico-striatal-thalamo-cortical (CSTC) loops (Alexander et al., 1986), anatomically segregated networks that mediate information transfer among functionally linked structures. CSTC loops modulate function beyond the cortex, striatum, and thalamus as well, through projections to the cerebellum, brainstem, and spinal cord (Haber, 2003). Identifying the afferent and efferent patterns of structural connectivity with each thalamic nucleus is essential to understanding the clinical impact of focal thalamic lesions (Hwang et al., 2017, 2020) and extrathalamic lesions that disrupt thalamic networks (Kim et al., 2021; Kletenik et al., 2022).The importance of CSTC loops in normal function and dysfunction in humans necessitates a more granular inspection of striatothalamic connectivity patterns (Joel, 2001).

Connectivity between the striatum and thalamus can be further specified based on the projections of two anatomically and functionally distinct compartments of the mammalian striatum, the striosome (aka patch) and matrix. Tract tracing studies in cats, rodents, and primates describe thalamostriate projections that are biased toward one striatal compartment (Nauta et al., 1974; Veening et al., 1980; Berendse and Groenewegen, 1990; Ragsdale and Graybiel, 1991; Féger et al., 1994). While thalamo-striate projections have been extensively mapped through tracer injections, and one study (Aoki et al., 2019) mapped multi-synaptic CSTC circuits using retrograde tracers, we found no prior reports that mapped compartment-specific multi-synaptic striato-pallido-thalamic projections. Therefore, the contributions of striosome and matrix to the regulation of specific thalamic nuclei, and specific CSTC loops, remain unknown.

While striosome and matrix are indistinguishable by routine histologic stains, the compartments can be readily identified using immunohistochemical methods in animal and human post-mortem tissue (Graybiel and Ragsdale, 1978; Holt et al., 1997). Both striatal compartments are comprised of medium spiny neurons (MSNs), but the two populations migrate from the lateral ganglionic eminence at different times in development (Graybiel and Hickey, 1982; Kelly et al., 2018). They have largely distinct patterns of afferent and efferent connectivity (Lévesque and Parent, 2005; reviewed in Waugh et al., 2022), segregated vascular supplies (Feekes and Cassell, 2006), and distinct expression of >60 surface proteins (Morigaki and Goto, 2015). Similar to other primate and non-primate mammals, in the human striatum the striosome is enriched in the medial, rostral, and ventral striatum – while the matrix is more common in the lateral, caudal, and dorsal striatum (Graybiel and Ragsdale, 1978; Goldman-Rakic, 1982; Donoghue and Herkenham, 1986; Graybiel, 1990; Desban et al., 1993; Eblen and Graybiel, 1995). They appear to fill opposing roles in behavior, with distinct influences on cost–benefit assessments, multiple models of learning, and motor action selection (Joel et al., 2002; Stephenson-Jones et al., 2013; Bloem et al., 2022).

We recently demonstrated that diffusion tensor imaging (DTI) methods can identify voxels with striosome-like and matrix-like patterns of connectivity in living humans (Waugh et al., 2022). Parcellating the striatal compartments based on differential structural connectivity replicated many anatomic features demonstrated through immunohistochemistry – their relative abundance, the anatomic sites where striosome or matrix are differentially enriched, their somatotopic organization, and their connectivity with extrastriate regions that were not utilized for parcellation. Test–retest reliability of this method was high: in humans scanned twice (1 month between scans), only 0.14% of voxels changed compartment-specific identity (Waugh et al., 2022). In regions where prior tract tracing studies in animals identified a robust compartment-specific bias in connectivity, our method replicated those findings in 93% of regions. An important limitation of this method is the size mismatch between the human striosome and a diffusion voxel, the unit of signal acquisition in our technique. We estimate that the diameter of the typical human striosome in coronal sections ranges from 0.5–1.25 mm [based on histology presented by Graybiel and Ragsdale (1978) and Holt et al. (1997)]. In contrast, the resolution of diffusion voxels in the present study was 1.5 or 2.0 mm isotropic, ensuring that even the most striosome-enriched striatal voxel will include a mix of striosome and matrix tissue. Readers should bear in mind that connection probabilities at each voxel are an average of both compartments, and that the majority of striatal voxels will therefore have indeterminate or weakly-biased compartment-specific connectivity profiles. For this reason, we restrict our assessments to the most-biased voxels, those that exceed 1.5 standard deviations beyond the mean for structural connectivity. Since our connectivity-based parcellations are inferential, we identify these voxels as “striosome-like” and “matrix-like” to remind readers that we have not directly identified striosome and matrix.

The organization of the striatum into striosome and matrix is conserved across all mammalian species investigated to date. Moreover, development of MSNs into striosome or matrix appears to be more fundamental than development of the direct and indirect pathways, a later-arising and less-distinct striatal organizational schema (Crittenden and Graybiel, 2011; Kelly et al., 2018). Projections from most cortical areas (80.0%) are significantly segregated toward one compartment (striosome-favoring or matrix-favoring, Waugh et al., 2022) suggesting that most CSTC loops follow a bifurcated path through striosome or matrix. While it is plausible that thalamic nuclei receive a combination of striosome-originating and matrix-originating projections, we hypothesize that the striato-pallido-thalamic projections to a specific thalamic nucleus may be biased toward one compartment. Striosome and matrix differ in many clinically-relevant ways: they have differential susceptibility to hypoxic–ischemic injury (Burke and Baimbridge, 1993) and to dopamine-mediated excitotoxicity (Saka et al., 2004; Granado et al., 2010; Jaquins-Gerstl et al., 2021); electrical stimulation promotes reward-mediated learning, but only when electrodes are placed in the striosome (White and Hiroi, 1998); dopaminergic D1 stimulation prolongs neuronal activation in matrix but shortens neuronal activation in striosome (Prager et al., 2020); and more than a dozen diseases have been hypothesized to have a compartment-specific neuropathology (Crittenden and Graybiel, 2011; Kuo and Liu, 2020). These observations suggest that characterizing compartment-specific biases in striato-pallido-thalamic projections may be important for understanding why diseases that affect the striatum or thalamus produce specific constellations of symptoms. To the best of our knowledge, such biases in compartment-level striatal projections to the thalamus as a whole, or to individual thalamic nuclei, have not been mapped previously in any species.

Much of our knowledge of the functions of thalamic nuclei in humans is based on correlation from animal studies, from observations in neurologic diseases [e.g., stroke (Schmahmann, 2003; Li et al., 2018), epilepsy (Leiguarda et al., 1992; Sánchez Fernández et al., 2012), traumatic brain injury (Snider et al., 2020; Mofakham et al., 2021], and from the response to focal treatments of neurological diseases, such as deep brain stimulation (DBS, Kundu et al., 2018; Morishita et al., 2019) and high intensity focused ultrasound (Shah et al., 2020). These studies demonstrate that specific thalamic nuclei, mechanisms of injury, and neuropsychiatric symptoms are linked, and that these thalamic dysfunctions can result from primary dysfunction within the thalamus or can result from extrathalamic sites impinging on the thalamus (Morton et al., 1993; Hedreen and Folstein, 1995; Chung et al., 1996; Miyai et al., 2000; Kumral et al., 2001). We hypothesize that the striosome and matrix, through their divergent roles in striatal function, may contribute to the specificity of thalamic nuclear functions. A non-invasive, *in vivo* assessment of compartment-specific thalamic connectivity may provide additional targets and more precise placement for neuromodulation (e.g., DBS), and may provide a nuanced understanding of complex brain injuries in which both striatum and thalamus are injured (Aravamuthan and Waugh, 2016).

In this study, we mapped the structural connectivity of thalamic nuclei with the striosome-like and matrix-like compartments of the striatum in living humans using probabilistic diffusion tractography. We utilized a relatively large (221 individuals) and racially diverse cohort of healthy adults, with equal representation of female and male subjects, and robust representation of ages from 20 to 65 years. This broad representation allowed us to assess for demographic influences on striato-pallido-thalamic connectivity. We cross validated our results against measures of structural connectivity in non-human primates, cats, and rats that used injected tract tracers. Our results suggest that the functional specialization of CSTC loops involves distinct paths through the striosome and matrix compartments of the striatum, and that these loops may be reliably assessed in living humans using probabilistic diffusion tractography.

## 2. Methods

### 2.1. Participants

All research was conducted in accordance with the principles set forth in the Declaration of Helsinki. We included subjects from multiple institutions. All data collection was approved by the Institutional Review Board for the respective institution where the subject was recruited. We utilized four separate cohorts of healthy subjects, totaling 221 participants (442 hemispheres). Of these 221 participants, 218 were determined to have connectivity data in at least one hemisphere that met our internal quality control assessments, and were therefore utilized for connectivity analyses (423 hemispheres). The pre-set criteria used to determine whether connectivity data was sufficient for inclusion is explained in section 2.6. We previously described the demographic variables of three of these cohorts (Waugh et al., 2022), which included 121 subjects (Cohorts 1–3). Briefly, 106 of these participants were right-handed (90%), 58 self-reported as female (49%), and the mean age of these cohorts was 35 years (range: 18–74). The remaining 100 subjects (Cohort 4, not previously described) were derived from the Human Connectome Project (HCP), accessed through the National Institute of Mental Health Data Archive (NDA, van Essen et al., 2013). We assembled a diverse cohort of HCP subjects, balancing subjects for age, sex, and self-identified race. To create the HCP cohort, we identified 10 HCP subjects (with a goal of five male, five female) at each five-year increment starting from 20 years old and ending at 65 years old (20, 25, 30, etc.). When there were insufficient numbers of HCP subjects at a particular age target, we selected subjects at surrounding ages, as close as possible to our target age, never infringing on the adjacent age targets. If there were fewer than five male or female subjects available at a specific age block, we rebalanced the sex ratio in another age block. For example, if only four male subjects were available at 30 years, we identified six female subjects at 30 years, and then at 35 years included six male and four female subjects. Among each group of five subjects (male or female, at a specific age), we attempted to produce a diverse and representative cohort by including at least one Asian subject, and at least one Black subject. We filled the remainder of each block of five with White subjects or those who listed their race as Other/Not reported. These participants were screened as healthy, with no reported history of neurological or psychiatric conditions. Of the 100 HCP subjects, the self-reported race was Asian (13%), Black (28%), White (52%), and Other/Not reported (7%). 90 of the HCP subjects were right-handed (90%) and 10 of the subjects were left-handed or ambidextrous (10%) based on the Edinburgh Handedness Inventory (Oldfield, 1971). The HCP cohort was evenly split between females and males (50:50). Subjects that comprise the HCP cohort can be accessed through the NDA via a study-specific identifier (DOI 10.15154/1528201). For our final, combined cohort (221 subjects), the mean age was 38.2 years old. The self-reported race of subjects was 39 Asian (22%), 37 Black (17%), 121 White (56%), and 10 Other/Not reported (5%). 113 participants were male (52%), and 105 were female (48%). Note that none of the included studies assessed gender identity; whether gender differed from the sex assigned at birth for any subject is unknown. The cohort included 196 right-handed subjects (90%), 21 left-handed or ambidextrous subjects (10%), and one subject who lacked handedness data.

### 2.2. Imaging data acquisition

All subjects were scanned at 3 T using whole-brain diffusion tensor imaging (DTI) and T1 (MPRAGE) protocols. For all subjects, MRI data was collected in a single scan session. We previously described the imaging acquisition for cohorts A, B, and C (Waugh et al., 2022). Briefly, these subjects were scanned at 2 mm isotropic resolution, using 70 direction (cohorts A and C, 10 B_0_ volumes and 60 volumes at non-colinear directions) or 33 direction (cohort B, one B_0_ volume and 32 volumes at non-colinear directions) DTI protocols. Data for HCP subjects was collected at three sites in the United States on Siemens Prisma scanners running Syngo MR E11 software and using harmonized protocols. Briefly, DTI for HCP subjects was acquired at 1.5 mm isotropic resolution using 200 directions (14 B_0_ volumes, 186 volumes at non-colinear directions) with the following parameters: repetition time = 3.23 s; echo time = 0.0892. Given these differences in DTI acquisition, we included both scanner type and number of diffusion directions as covariates in our subsequent analyses. T1 scans were collected at 1 mm isotropic resolution for cohorts A–C, and 0.80 × 0.76 × 0.76 mm for HCP subjects. The imaging volumes acquired for Cohorts A, B, C, and HCP were: A, 256x256x256mm; B, 180 × 240 × 240 mm; C, 128 × 128 × 128 mm; HCP, 208 × 300 × 320 mm. We utilized the Freesurfer utility *recon-all* to perform whole-brain segmentation. Regardless of the original resolution and imaging volume for each subject, *recon-all* standardized the resolution of all T1 images to 1 mm isotropic.

### 2.3. Thalamic parcellations

We utilized the automated method of Iglesias et al. (2018) to produce individualized segmentations of the thalamus in native T1 space. Briefly, we completed whole brain segmentation using *recon-all* (Reuter et al., 2012), utilizing each subject’s MPRAGE scan. We visually inspected the results of *recon-all* for each subject. Next, we utilized the Iglesias add-on to *recon-all* to further segment the thalamus into 25 nuclei. We planned to use these thalamic segmentations to extract connectivity estimates from probabilistic tractography (after registering tractographic probability maps into native T1 space) but recognized that image registration and partial volume inaccuracies might compromise accurate data extraction. Therefore, we established, *a priori,* a minimum volume threshold for including data from a thalamic nucleus. We set a lower volume limit of four DTI-space voxels (32mm^3^ for cohorts A–C; 13.5mm^3^ for our HCP cohort) when registered into T1 space, which excluded seven nuclei for cohorts A–C: central lateral, laterodorsal, limitans (suprageniculate), reuniens (medial ventral), paracentral, paratenial, and ventromedial. Note that the thresholds of 32mm^3^ for cohorts A–C and the 13.5mm^3^ for our HCP cohort are actually the same voxel-based threshold – four native-space voxels, adjusted for the resolution of each diffusion protocol. In addition, we excluded the lateral geniculate and limitans nuclei, given that (1) the white matter architecture surrounding these nuclei made grey/white segregation and accurate segmentation inconsistent in our datasets, and (2) we could identify no prior animal literature documenting afferent or efferent projections between the striatum and either nucleus.

Our exclusion criteria left 17 thalamic nuclei for final analysis in all 221 subjects: anteroventral (AV), central medial (CeM), centromedian (CM), lateral posterior (LP), mediodorsal-lateral (MDl), mediodorsal-medial (MDm), medial geniculate (MGN), parafascicular (Pf), pulvinar-anterior (PuA), pulvinar-inferior (PuI), pulvinar-lateral (PuL), pulvinar-medial (PuM), ventral anterior (VA), ventral anterior magnocellular (VAmc), ventral lateral anterior (VLa), ventral lateral posterior (VLp), and ventral posterolateral (VPL). Given the higher resolution of DTI scans in our HCP cohort (1.5 vs. 2.0 mm isotropic), three additional nuclei met volumetric criteria when assessed only in the HCP cohort: central lateral (CL), laterodorsal (LD), and reuniens (Reu). We therefore assessed CL, LD, and Reu only in the 100 subjects of the HCP cohort.

### 2.4. DTI processing

Our A, B, and C cohorts had only anterior-to-posterior imaging, whereas the HCP group had both anterior-to-posterior and posterior-to-anterior DTI volumes. Therefore, only HCP subjects were eligible for susceptibility-induced distortion correction using the FSL utility *topup*. We performed skull stripping using the FSL Brain Extraction Tool (*bet2*). We utilized the FSL tool *eddy* to correct for eddy current-induced distortions and subject motion. We fit local diffusion tensors using *dtifit*, creating a 3D FA image at the same resolution as the original diffusion images. Finally, we generated diffusion parameters at each voxel using *bedpostx* (Behrens et al., 2007). We completed preprocessing and probabilistic tractography steps in each subject’s native space.

### 2.5. Probabilistic diffusion tractography

We carried out four separate iterations of probabilistic tractography for this study, each with a distinct purpose. *First*, we completed striatal parcellation using classification targets tractography (CTT) to quantify structural connectivity, as we described previously (Waugh et al., 2022). Briefly, we utilized a series of striosome-favoring or matrix-favoring cortical “bait” regions to identify voxels whose patterns of connectivity with extra-striate targets matched those identified in animals – and thus were “striosome-like” or “matrix-like.” We identified these bait regions from our comprehensive review of prior tract tracing studies in animals (Waugh et al., 2022). Unlike traditional probabilistic tractography, which focuses on streamlines after they exit a seed voxel, CTT quantifies the structural connectivity at each seed voxel with a predefined set of targets. Therefore, the output of CTT is a series of probability distributions – in this case, two superimposable maps of the striatum, one measuring connectivity to striosome-favoring regions, the other measuring connectivity to matrix-favoring regions. The ratio of those two probability distributions quantifies the degree to which a voxel is connected in a striosome-like or matrix-like manner. *Second*, we executed traditional streamline tractography (non-CTT) to quantify and localize the streamline bundles that connect striosome-like and matrix-like striatal voxels with the thalamus. *Third*, we performed CTT with the thalamus as seed and the sets of parcellated striosome-like and matrix-like voxels as competing targets (the second round of CTT described here). We used this third iteration of tractography to quantify connectivity between specific thalamic nuclei and each striatal compartment. *Fourth*, we conducted two *post-hoc* iterations of striatal parcellation to assess the importance of precision in selecting striatal voxels. These parcellations utilized “N-1” (aka “leave-one-out”) approaches, in which we used five matrix-favoring regions as bait, but only four striosome-favoring regions as bait. This allowed us to quantify connectivity with the left-out region, as we demonstrated previously (Waugh et al., 2022). For all four iterations of tractography, we ran left and right hemispheres independently. Our striatal parcellations (tractography iteration one) and post-hoc assessments of precision (tractography iteration four) utilized a whole-hemisphere bounding mask. Iterations two and three utilized a subcortical bounding mask, described below.

The bait regions we utilized for striatal parcellation were similar to those we described previously (Waugh et al., 2022). However, as the present analysis included the globus pallidus interna (GPi) and thalamus as waypoint or seed regions, respectively, we could not utilize these regions in striatal parcellation – one cannot define striatal compartment identity based on connectivity to a region and subsequently quantify connectivity with that region. Therefore, we replaced three bait regions from our prior work with three cortical regions whose striatal connectivity was significantly biased toward one compartment: (i) superior parietal (matrix-favoring), (ii) the superior portion of the inferior frontal gyrus, pars opercularis (matrix-favoring), and (iii) the posterior portion of the temporal fusiform cortex (striosome-favoring). We selected these cortical regions for their high degree of compartment selectivity and substantial reproducibility across imaging cohorts. However, we were unable to identify any prior tract tracing studies in animals that mapped connectivity with these three regions. Notably, the temporal fusiform cortex is present only in hominids, and the superior parietal lobule and inferior frontal gyrus are present only in primates, precluding their study in the animal species commonly utilized in tract tracing studies (Waugh et al., 2022). We aimed to validate this group of bait regions by assessing whether selecting striatal voxels based on biased connectivity would reproduce other features of the striatal compartments demonstrated through histology, such as the relative location of each compartment within the striatum. Striosome and matrix are not distributed randomly within the striatum [both in animal (Goldman-Rakic, 1982; Gerfen, 1984) and human histology (Graybiel and Ragsdale, 1978; Faull et al., 1989)]. We tested the intra-striate position of striosome-like and matrix-like voxels to learn whether identifying voxels based on biases in connectivity could also match the spatial patterns expected from histology. For the most-biased voxels in the striosome-like and matrix-like distribution (those used as the seed or targets of subsequent rounds of tractography), we measured their cartesian location relative to the centroid of the nucleus it occupied (left or right hemisphere, caudate or putamen).

All standard space regions of interest (ROI) utilized in this study are provided in our Supplemental Materials. These standardized masks, when registered into a subject’s native diffusion space, served as seed, waypoint, target, inclusion, or exclusion masks for probabilistic diffusion tractography, which we carried out with the FSL tool *probtractx2* (Behrens et al., 2007). We used *fslview* to manually segment left and right thalamic masks, encompassing only the thalamus and excluding the surrounding white matter. Our striatal ROI did not include the nucleus accumbens, which does not share the striosome/matrix architecture observed in the rest of the striatum, and excluded the posterior half of the caudate tail, as we found that this small structure led to registration errors and frequent partial volume effects (Waugh et al., 2022). We generated a subcortical inclusion mask, which encompassed the caudate, putamen, globus pallidus (interna and externa), thalamus, and the white matter immediately surrounding these structures. This mask eliminated all streamlines that extended beyond its boundaries, excluding corticostriate, thalamocortical, and subcortical-brainstem projections. We utilized this subcortical inclusion mask to refine tractography iterations two and three (mapping striato-pallido-thalamic streamline bundles, and quantifying connectivity at the level of specific thalamic nuclei, respectively). Seven of the cortical ROI masks used for striatal parcellations and our GPi mask were defined in our prior work (Waugh et al., 2022). We segmented the three new cortical ROIs on the MNI152_T1_1mm standard brain utilizing the human brain atlas of Mai et al. (1997) based on prior MRI assessments of the superior parietal (Passarelli et al., 2021), fusiform (Rajimehr et al., 2009), and inferior frontal (Hammers et al., 2007) gyri. We registered all standard-space ROIs into each subject’s native space using the FSL tools *flirt* and *fnirt*.

The results of probabilistic tractography can be influenced by the size of the target masks utilized, with larger volume increasing the probability of any given streamline ending at the target mask. Therefore, we assured that striosome-like and matrix-like masks from the same hemistriatum always had equal volume in order to minimize this source of bias, as previously described (Waugh et al., 2022). To create these matched-volume striatal masks, we selected the N most-biased voxels in each probability distribution (striosome-like and matrix like), retaining the voxels that were 1.5 standard deviations above the mean. Since our experimental cohorts included diffusion images with two different resolutions, we set two different volume thresholds to match this 1.5 standard deviation target. For cohorts A–C (2 mm isotropic voxels), we utilized an 83 voxel threshold. For our HCP cohort (1.5 mm isotropic voxels), we utilized a 180 voxel threshold. Striosome-like and matrix-like voxels cannot overlap; our method for selecting biased voxels precludes the same voxel being selected to represent both compartments. The overlap between compartment-like voxels within a subject was zero.

We utilized these equal-volume striatal compartment masks in tractography iterations two and three; iterations one and four (striatal parcellation) utilized the whole-striatum mask. For tractography iteration one (CTT, striatal parcellation), we utilized the striatum as seed, 10 cortical ROIs (5 striosome-favoring, 5 matrix-favoring) as targets, and included streamline paths from the whole hemisphere. For tractography iteration two (mapping the path of striato-pallido-thalamic streamlines) we utilized equal-volume striatal masks (striosome-like or matrix-like) as seeds, the GPi as a waypoint (streamlines were rejected if they did not contact the GPi), and the thalamus as a termination mask (streamlines terminated upon contacting any thalamic voxel, preventing loopbacks). For tractography iteration three (CTT, quantifying connectivity with each thalamic nucleus) we utilized the thalamus as seed, GPi as waypoint, and striosome-like and matrix-like masks as targets. Note that since the streamlines of probabilistic tractography have no directionality, this round of CTT selected for the same striato-pallido-thalamic streamlines generated using the traditional streamline tractography (iteration two) but allowed us to quantify connectivity at each thalamic voxel.

For tractography iteration four (striatal parcellation followed by quantitative CTT), we parcellated the striatum as described for iteration one, but used 9 cortical ROIs (4 striosome-favoring, 5 matrix-favoring) as targets. We carried out this N-1 parcellation twice, once each for two regions that were among the strongest striosome-favoring biases from our prior work (Waugh et al., 2022): anterior insula and basal operculum. We then performed quantitative CTT with these left-out regions as seed and the N-1 parcellated striatal voxels as targets. As our aim for this iteration of tractography was to assess the influence of precise voxel location on compartment-level bias, we ran parallel versions of quantitative CTT with imprecise striatal voxels as targets. To generate these imprecise masks, we shifted the position of each voxel in our precise, matched-volume masks, at random, by +/− 0–3 voxels in each plane. Note that this randomization step shifted the location by only a few voxels – these imprecise voxels were near-neighbors of the precise matched-volume masks. We measured the amplitude of this location shift for 40 hemispheres, selected at random: five striosome-like and five matrix-like masks for each of our four experimental cohorts. We measured the change in location (root-mean-square distance) for all 4,026 voxels in these 40 hemispheres.

We completed striatal parcellation and mapped the path of striato-thalamic streamlines (tractography iterations one and four, and two, respectively) using the standard *probtrackx2* settings: curvature threshold = 0.2; steplength = 0.5 mm; number of steps per sample = 2,000; number of streamlines/voxels = 5,000. For quantifying striato-thalamic tractography at the level of individual thalamic nuclei, however, increasing the depth of sampling by 10× (50,000 streamlines/voxel) yielded a more accurate probability distribution. Specifically, increasing the number of streamline trials provided a more robust mapping of each compartment’s connection probability and thereby decreased the number of thalamic voxels that had no streamlines that met our anatomic and tractographic criteria. This increase in streamlines/voxel reduced the impact of “floor effects,” locations where we otherwise could not distinguish between low-connectivity and no-connectivity voxels. This allowed connectivity estimates to derive from a larger fraction of the voxels in each thalamic nucleus. For the MGN, we found that a further increase to 500,000 streamlines per seed voxel was necessary to adequately sample the probability distribution. Therefore, we ran tractography and quantification for MGN separately from all other thalamic nuclei. In all three iterations of probabilistic tractography, streamlines were corrected for length to prevent the proximity to a target from influencing the strength of connection. We visually inspected the results of *dtifit*, *bedpostx,* native space registrations, and both CTT and streamline tractography, for each subject, to assure that DTI processing was complete and accurate.

The regional segmentations and Linux scripts utilized to parcellate the striatum are accessible here: github.com/jeff-waugh/Striatal-Connectivity-based-Parcellation.

### 2.6. Localizing compartment-specific streamlines

Our goals for tractography iteration two were (1) to map the paths of striato-pallido-thalamic connectivity, and (2) determine the relative abundance of streamlines seeded by striosome-like vs. matrix-like voxels. We sought to determine whether the streamlines seeded from striosome-like and matrix-like voxels traversed different routes to reach their pallidal and thalamic targets. Therefore, we utilized normalized streamline bundles to reduce the impact of tract amplitude on mean location: we divided each subject/hemisphere/compartment’s tractography volume by its maximum value within the GPi. The averaged images and *randomise* testing results discussed below utilized these normalized tracts. After registering each subject’s tractography into standard space, we used two distinct approaches to assess the overlap of streamlines seeded by striosome-like vs. matrix-like voxels. First, we used *fslstats* to determine the site of maximum value of striato-pallido-thalamic projections within the GPi (independent striosome-like and matrix-like streamline bundles). We quantified the location difference between compartment-specific bundles by calculating the root-mean-square difference in the site of the peak streamline amplitude (striosome-like vs. matrix-like bundles, in each individual and hemisphere). Further, we compared the x-, y-, and z-plane coordinates of these maximum value sites between striosome-like and matrix-like bundles in each individual and hemisphere. To correct for the intrinsic differences in location of the GPi in left and right hemispheres, we matched the center of mass for left and right GPi by mirroring right hemisphere x-coordinates into the left hemisphere and bringing y- and z-coordinates to the mean y- and z-coordinates for left and right GPi. Second, we quantified the overlap in streamlines using the averaged striato-pallido-thalamic tractograms from all subjects, for each hemisphere and compartment-specific seed mask, both within and outside the GPi. For each hemisphere and compartment-specific seed we isolated the core of the tract at high- (uppermost 25% of voxels by amplitude), mid- (uppermost 50%), and low-stringency (uppermost 90%) amplitude thresholds. We previously identified these high- and mid-stringency thresholds as sufficient to isolate the tract core (Waugh et al., 2019). We then calculated the Dice Similarity Coefficient (DSC) at high-, mid-, and low-stringency to assess the overlap in striosome-like and matrix-like streamlines, both within and outside the GPi, for each hemisphere. We then quantified the overlap of streamlines for individual subjects with the mean streamline bundle (uppermost 50%) to assess location variance within each streamline bundle. Finally, we assessed the number of streamlines per seed voxel (non-normalized), comparing streamline bundles seeded by striosome-like vs. matrix-like voxels.

### 2.7. Quality assurance and quantification of thalamic connectivity

We registered each subject’s thalamic probability maps (tractography iteration three) into native T1 space. This allowed us to utilize each individual’s *recon-all* thalamic segmentation to generate nucleus-specific masks for extracting connectivity data from striosome-like and matrix-like probability distributions. For native space probability maps, at every voxel the striosome-like and matrix-like probability distributions summed to one. After registration to T1 space, partial volume and edge effects led some voxels to lose this “sum to one” property. Therefore, we renormalized the probability distributions on a voxel-by-voxel basis. We trimmed edge voxels whose summed value was <0.5, which reduced partial volume effects at the edges of the thalamus. For each thalamic nucleus, we quantified the number of suprathreshold voxels (value ≥0.55) in striosome-favoring and matrix-favoring normalized probability distributions. For each nucleus we expressed connectivity as the percent of suprathreshold voxels dominated by each compartment (N_voxels_, striosome-like or matrix-like)/(N_voxels_, striosome-like + N_voxels_, matrix-like).

Next, we assessed each thalamic nucleus for connectivity differences between left and right hemispheres. For any nucleus whose compartment-specific connectivity (1) was significantly different between left and right hemispheres and (2) was biased toward different compartments in the two hemispheres, we reported results for left and right hemispheres independently. For the four nuclei that met these criteria, bias was significant in one hemisphere and neutral in the other; we did not find significant differences in compartment-by-hemisphere bias (e.g., left hemisphere biased toward matrix-like, and right hemisphere biased toward striosome-like voxels) for any thalamic nucleus. For the remaining 16 nuclei we combined left and right hemispheres for quantifying striosome-like vs. matrix-like patterns of connectivity.

Prior to any statistical comparisons, we imposed a data quality threshold to reduce inaccuracies resulting from inadequate sampling of the thalamic probability distributions. First, we summed the volume of suprathreshold voxels from the striosome-favoring and matrix-favoring probability distributions (the maps resulting from whole-thalamus CTT). As we were concerned that paucity of data might reduce the accuracy of quantification, we eliminated all subject-hemispheres whose suprathreshold total volume was below 10% of the mean volume for that hemisphere. This elimination removed the left hemisphere for 13 participants and the right hemisphere for 6 participants. Next, we eliminated all subject-hemispheres that had no data for one compartment, as this led to a binarization of data that skewed bias calculations. For example, two subjects with very different bias counts would result in the same volume percent calculation (subject 1: 0 matrix-favoring voxels, 99 striosome-favoring voxels; subject 2: 0 matrix-favoring voxels, 1 striosome-favoring voxel; both result in bias estimates of 100% striosome-favoring). Note that increasing the streamlines/voxel during CTT (from 5,000 to 50,000 per seed voxel) substantially reduced the number of under-sampled subject hemispheres. Following this quality control step, we assessed thalamic connectivity in 210 left and 214 right hemispheres. For all subject-hemispheres eliminated based on under-sampling of thalamic CTT (tractography iteration three), we also removed those subjects from assessments of tractography iteration two.

Finally, we compared our MRI-based results with previously published tract tracing studies in animals. For every prior study, we recorded the tracing methods, species utilized, the number of animals assessed, and whether compartment assessment directly visualized tracing material in either striosome or matrix, or indirectly suggested compartment-specific connectivity. We noted compartment-specific assessments as-described by the original authors; we did not reinterpret findings.

### 2.8. Statistical analyses

All statistical tests on data extracted from tractography volumes were performed using STATA (van Essen et al., 2013, Stata Statistical Software: Release 13, College Station, TX). We performed voxelwise nonparametric permutation testing on tractography volumes using the FSL tool *randomise*. We measured the location of each voxel in our striosome-like and matrix-like masks (the cartesian position relative to the centroid of caudate or putamen) for each subject and hemisphere, producing a dataset of 103,688 parcellated voxels. We assessed the effect of striatal compartment and nucleus of origin (caudate or putamen) on voxel location using two-factor ANOVAs (one each for the x-, y-, and z-planes). Within the same ANOVAs we assessed the effect of cohort, and subject identity nested within cohort. To the best of our knowledge, no interhemispheric differences in matrix and striosome location have been described previously. Therefore, we did not treat hemisphere as a separate factor. We previously demonstrated that scanner type, subject sex, and self-identified race had no influence on the location of parcellated striatal voxels (Waugh et al., 2022), and we therefore did not include these factors in our model. We performed identical ANOVAs for the x-, y-, and z-planes, and therefore used a significance threshold of *p* < 0.017 (0.05/3 comparisons). We performed post-hoc analyses of simple main effects for all factor interactions using the SME utility developed by the UCLA ATS Statistical Consulting Group (Ender, 2017). We utilized the simultaneous test procedure for estimating the critical value of F, the most conservative method provided in this utility.

We evaluated the influence of precise location on compartment-level bias by comparing the results of CTT with precisely-selected versus neighboring (imprecise) striatal voxels. We performed paired samples t-tests for the mean probability of connection, volume projecting to striosome-like voxels, and volume projecting to matrix-like voxels, for two striosome-favoring seed regions, the anterior insula and the basal operculum. We therefore used a significance threshold of *p* < 8.3×10^−3^ (0.05/6 comparisons).

We utilized paired-samples t-tests to compare the location of the peak value within the GPi for streamline bundles that originated in striosome-like or matrix-like voxels. As we tested location in the x-, y-, and z-planes, we utilized a significance threshold of *p* < 1.7×10^−2^ (Bonferroni correction for three tests, 0.05/3). We utilized a single paired-samples t-test to compare the number of streamlines per voxel originating from striosome-like and matrix-like voxels. Our significance threshold was *p* < 0.05. We compared thalamic nucleus-specific connectivity in the left and right hemispheres using *t*-tests, two samples with equal variance. As we tested 20 thalamic nuclei for hemispheric differences, our significance threshold was *p* < 2.5×10^−3^ (Bonferroni correction for 20 tests, 0.05/20). We performed a post-hoc series of ANCOVAs to identify any subject factors that were drivers of interhemispheric difference. Factors assessed included age, number of diffusion directions, scanner type, handedness, hemisphere, self-identified race, and sex. As Cohorts A and B were unbalanced for sex, and Cohort B was also unbalanced for self-identified race, we also assessed the interaction between sex, self-identified race, diffusion directions, and scanner type. Data from different thalamic nuclei was colinear, precluding our use of MANCOVA to combine these tests. Therefore, we performed 20 independent ANCOVAs, resulting in a significance threshold for these ANCOVAs of *p* < 2.5×10^−3^ (Bonferroni correction for 20 tests, 0.05/20). We assessed compartment-specific bias in each thalamic nucleus using *t*-tests, two samples with equal variance. As we measured the bias in combined hemispheres in 16 nuclei, our significance threshold for these comparisons was *p* < 3.1×10^−3^ (Bonferroni correction for 16 tests, 0.05/16). Finally, we performed voxelwise nonparametric permutation testing of striato-pallido-thalamic streamlines (*randomise*) with the following parameters: 5,000 permutations; variance smoothing = 2 mm; threshold-free cluster enhancement mode; masked by one of two conditions in separate iterations of *randomise*. The two masks utilized for *randomise* were (1) the same GPi mask used as a waypoint for tractography, or (2) the subcortical bounding mask utilized for tractography with all grey matter structures removed (to restrict testing to only the subcortical white matter). For both iterations of randomise, we used the familywise error corrected, threshold-free cluster enhanced test statistics. Since we performed two related tests using randomise, we set our significance threshold at *p* < 0.025 (0.05/2).

## 3. Results

### 3.1. Overview of experimental aims

We aimed to identify the thalamic nuclei whose striato-pallido-thalamic structural connectivity was significantly biased toward striosome-like or matrix-like voxels – the striatal voxels whose corticostriate connectivity profiles matched the biases demonstrated in prior tract tracing studies in animals. These striatal parcellations are highly stable: in subjects scanned twice, with 1 month between scans, only 0.14% of striatal voxels switched compartment identity (Waugh et al., 2022). We first measured the locations of our parcellated striatal voxels to assure that our method identified voxels with striosome-like and matrix-like distributions within the striatum, in addition to their striosome-like and matrix-like patterns of connectivity. We next assessed the precision of our striatal parcellations by comparing quantitative tractography when striatal target voxels were either precisely selected or were randomly selected from among the nearest neighbors of those precise voxels. Then, we assessed whether our tractographic approach produced distinct and anatomically plausible streamline bundles by quantifying streamline amplitude and intra-pallidal location of streamline bundles. Next, for all thalamic nuclei whose volume was sufficient to allow for reliable assessment, we tested whether thalamic nuclei in the left and right hemispheres had the same or different biases in striato-pallido-thalamic connectivity. Finally, we evaluated each of these thalamic nuclei for compartment-specific biases in structural connectivity.

### 3.2. Compartment-specific voxel location

Prior histologic studies in humans (Graybiel and Ragsdale, 1978; Faull et al., 1989; Holt et al., 1997) and animals (Graybiel and Ragsdale, 1978; Goldman-Rakic, 1982; Gerfen, 1984; Donoghue and Herkenham, 1986; Malach and Graybiel, 1986) described medio-lateral, rostro-caudal, and dorsal-ventral gradients in the predominant locations of each compartment. Though striosomes can be found throughout the striatum, they are enriched in medial, rostral, and ventral sites. Our connectivity-based parcellation method identified a similar pattern: striosome-like voxels are located more medial, more rostral, and more ventral than matrix-like voxels. Two-factor ANOVA, examining the effects of striatal compartment and striatal nucleus (caudate or putamen), identified a significant influence of both factors, and their interaction, on voxel location.

The mean location of striosome-like voxels was 0.9 mm more medial (F[1, 223] = 2,668, *p* < 4.6×10^−126^), 4.1 mm more rostral (F[1, 223] = 8,468, *p* < 2.3×10^−179^), and 4.9 mm more ventral (F[1, 223] = 44,277, *p* < 1.9×10^−258^) than the mean location of matrix-like voxels. Whether a voxel was found within the caudate or putamen also had a significant influence on voxel location. Matrix-like voxels in the putamen were more lateral (F[1, 223] = 90.8, *p* < 2.8×10^−18^), more caudal (F[1, 223] = 38.8, *p* < 2.3×10^−9^), and more dorsal (F[1, 223] = 1,309.9, *p* < 2.6×10^−95^) than matrix-like voxels in the caudate. Note that since individual voxels were assessed relative to the centroid of either caudate or putamen, the relative positioning of each nucleus within the hemisphere did not drive these differences in location. However, differences in the geometry and size of the caudate and putamen may have allowed voxels to reside at greater distance from the centroid of the putamen.

The interaction of compartment and nucleus had a significant effect on voxel location in the x-plane (sagittal, F[1, 223] = 1,084.7, *p* < 1.3×10^−87^), y-plane (coronal, F[1, 223] = 53.9, *p* < 3.9×10^−12^), and z-plane (axial, F[1, 223] = 15.6, *p* < 1.1×10^−4^). Simple main effects analysis of the compartment-nucleus interaction showed that in the x-plane, only putamen has a significant effect on voxel location (F[1, 103,464] = 7,667.3, *p* < 1.9×10^−305^). In the y- and z-planes, both caudate and putamen had a significant effect on voxel location: y {caudate, (F[1, 103,464] = 1,519.8, *p* < 1.1×10^−153^)}; putamen, (F[1, 103,464] = 10,205.5, *p* < 7.0×10^−321^); z {caudate, (F[1, 103,464] = 15,341.4, *p* < 2.7×10^−322^)}; putamen, (F[1, 103,464] = 40,976.3, *p* < 5.4×10^−322^). Variance from individual subjects and experimental cohort factors (Supplemental Figure 1) were not significant contributors to voxel location in any plane.

### 3.3. Compartment-specific bias depends on precise selection of striatal voxels

Biases in compartment-specific connectivity were highly influenced by precise voxel location. We performed N-1 parcellation, leaving out either the anterior insula or basal operculum in successive iterations of CTT, which allowed us to then perform quantitative tractography with each of these two striosome-favoring regions in turn. After generating matched-volume masks for each N-1 parcellation (the precise voxels with the highest degree of striosome-like or matrix-like bias in connectivity), we created parallel, imprecise masks with the location randomly shifted by up to 3 voxels in each plane. We sampled these random shifts in location for 40 hemispheres (4,026 voxels relocated). For both striosome-like and matrix-like masks, this randomization step led to a mean shift in location of 1.8 voxels (SEM, ±0.01 voxel). Importantly, these relocated voxels were among the nearest neighbors of our precisely selected voxels. In our control conditions (anterior insula or basal operculum as seed, precise striatal masks as target), both regions were highly biased toward striosome-like voxels. Of seed voxels with substantial bias (those with compartment-specific connection probability ≥0.87), 82.1% (SEM, ±1.2%) of anterior insular voxels and 91.5% (SEM, ±0.95%) of basal opercular voxels favored striosome-like target voxels. When precise compartment-like targets were shifted to neighboring voxels, the mean volume of anterior insula that favored striosome-like targets fell by 5.1-fold (from 1,438 to 279 voxels; *p* < 2.0×10^−118^; Supplemental Figure 2). Similarly, basal opercular voxels that favored striosome-like targets fell by 3.6-fold (from 1,213 to 339 voxels; *p* < 1.3×10^−111^). Targeting less-precise striatal voxels also led to an increase in the percentage of seed voxels that favored matrix-like connectivity, increasing by 19.0% in the anterior insula (*p* < 3.0×10^−3^) and 49.3% in the basal operculum (*p* < 3.6×10^−4^). Mean connectivity bias within the seed regions was also substantially reduced by shifting precise targets to neighboring voxels. The probability of connection to striosome-like voxels reduced by 35.8% in the anterior insula (*p* < 1.0×10^−101^) and reduced by 54.8% in the basal operculum (p < 2.7×10^−35^) when striatal targets were imprecise. Relocating striosome-like and matrix-like targets even 1–2 voxels from their precise location markedly reduced compartment-specific biases in connectivity.

### 3.4. Streamlines seeded by striosome-like and matrix-like voxels rarely colocalize

**Figure 1:**
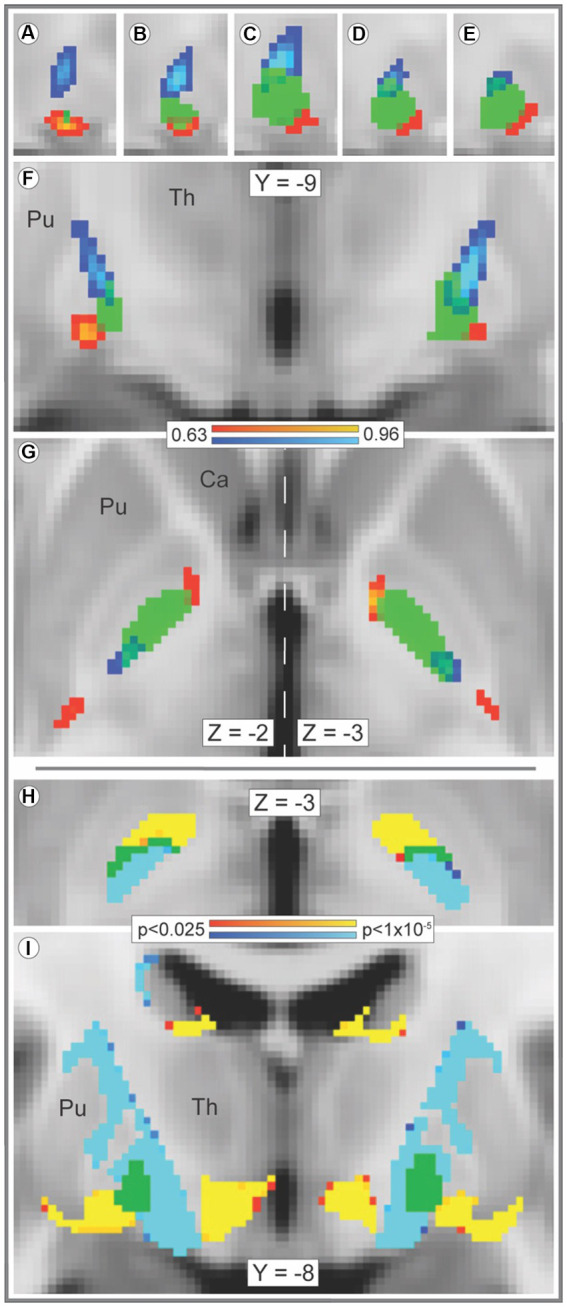
Streamline bundles originating in striosome-like or matrix-like voxels reach and traverse the GPi by different paths. Tracts seeded by matrix-like or striosome-like striatal voxels follow distinct paths through the globus pallidus interna (GPi). The averaged streamlines (**A–G**, range: 0.63–0.96) for all subjects are visualized in red-yellow (striosome-like seeds) and blue-light blue (matrix-like seeds) overlaid by the GPi mask (green). The GPi mask is shown at 50% opacity to visualize overlap with streamline bundles. In the x-plane (panels **A–E**), views of the right GPi (from x = 23–19) demonstrate segregation of the two streamline bundles at all lateral-to-medial (left to right) points. The bundles remain segregated in the y- **(F)**, and z-planes **(G)** as well. The locations of peak amplitude for averaged matrix-like and striosome-like streamlines had significantly different locations in each plane (x plane, *p* < 1.1×10^−35^; y plane, *p* < 1.3×10^−53^; z plane, *p* < 3.8×10^−42^). The core of streamline bundles that originated in matrix-like and striosome-like voxels (the uppermost 25% of each bundle, by amplitude) do not overlap in any GPi voxel. Voxelwise significance testing with *randomise* **(H,I)** demonstrated this segregation of streamlines seeded by striosome-like and matrix-like voxels as well (significance threshold, *p* < 0.025, corrected for multiple comparisons; visualized range: 0.025–0.00001). Bundles seeded by striosome-like voxels (red-yellow) and matrix-like voxels (blue-light blue) occupy distinct zones of the GPi **(H)** and the subcortical white matter **(I)**. In H, green voxels illustrate the few pallidal voxels where bundles were not significantly different. In **(I)**, green voxels illustrate the whole of the GPi, as significance testing in this panel included only white matter voxels. Optimal visualization in the z-plane **(G)** was offset by 1 mm in the left and right hemispheres (vertical dashed line). Images follow radiologic convention. Coordinates follow MNI convention. Ca, caudate; Pu, putamen; Th, thalamus.

Streamline bundles originating in striosome-like and matrix-like voxels occupied distinct locations within the GPi and approached the GPi from different orientations (Figure 1). This GPi organization was qualitatively symmetric between hemispheres (Figures 1F–I). The maximum values of bundles from the two compartments had significantly different locations in the x-, y- and z-planes (*p* < 1.1 × 10^−35^, 1.3 × 10^−53^, and 3.8 × 10^−42^, respectively), with streamlines seeded by striosome-like voxels situated more rostrally, ventrally, and laterally. Intra-pallidal location was highly similar within each bundle: coefficients of variation (CVs) for striosome-like streamlines (x-, y-, and z-planes) were 3.8, 2.6, and 2.9%, respectively; for matrix-like streamline locations, CVs were 1.8, 1.8, and 3.1%, respectively. The root-mean-square difference in the sites of peak value (striosome-like vs. matrix-like) within the GPi was 4.6 mm (SEM ± 0.089). Intra-pallidal streamlines in the core of the striosome-like and matrix-like bundles (the uppermost 25% of the amplitude distribution, Waugh et al., 2019) did not overlap in either hemisphere (DSC, 0%). At a mid-stringency threshold (the uppermost 50% of the amplitude distribution), streamlines were segregated (DSC, left GPi: 15.3% overlap; right GPi: 13.7% overlap). At a low-stringency threshold (the uppermost 90% of the amplitude distribution), streamlines were still largely segregated (DSC, left GPi: 34.1% overlap; right GPi: 32.8% overlap). Comparison of streamlines at the voxelwise level (with *randomise*, Figure 1H) yielded similar results: for the majority of pallidal voxels (left hemisphere, 83.0%; right hemisphere, 79.0%), streamline counts seeded by one compartment were significantly greater than those seeded by the other compartment, in a similar pattern demonstrated through DSC.

Striato-pallido-thalamic streamline bundles seeded from striosome-like and matrix-like voxels arrived at and occupied distinct parts of the GPi (Figures 1A–G,I). A dense band of streamlines that originated in matrix-like voxels reached the dorsoposterior GPi. Lower density bands of streamlines that originated in striosome-like voxels approached from rostral and caudal orientations; both approached the ventrolateral GPi. Streamlines were highly segregated outside the GPi as well. Streamlines from the uppermost 25% of the amplitude distribution did not overlap, in either hemisphere (DSC, 0%). Streamline bundles from the uppermost 50% of the distribution were highly segregated (DSC, left hemisphere: 2.7% overlap; right: 4.4% overlap). Even at a low-stringency amplitude threshold (the uppermost 90% of the distribution), extra-pallidal streamlines were largely segregated (DSC, left hemisphere: 31.0% overlap; right: 41.4% overlap). Among our 221 subjects, compartment-specific streamline bundles were highly colocalized: the core of each individual’s streamline bundle overlapped with the core of the average streamline bundle in 95.7 and 93.9% of our subjects (streamlines seeded by matrix-like and seeded by striosome-like voxels, respectively).

### 3.5. Abundance of compartment-specific streamlines reaching the GPi

Streamlines seeded from matrix-like voxels were 5.7-fold more likely to reach the thalamus (*via* the GPi) than streamlines seeded by striosome-like voxels (2,291 streamlines per matrix-like seed voxel vs. 401 streamlines per striosome-like seed voxel; *p* < 8.7×10^−31^). Within the GPi, streamlines seeded by matrix-like voxels were 7- to 9-fold more abundant than streamlines seeded by striosome-like voxels (left matrix-like: 727.3, SEM ±73.3; left striosome-like: 79.2, SEM ±27.1; right matrix-like: 536.6, SEM ± 89.8; right striosome-like: 70.0, SEM ± 14.3). Note that for each subject and hemisphere, striosome-like and matrix-like seed masks always had equal volume (the uppermost 1.5 standard deviation of each probability distribution) and were seeded with equal numbers of streamlines. Therefore, streamline counts were independent of the relative volume of striosome-like and matrix-like voxels in the whole striatum. The dominance of matrix-like voxels in the GPi is consistent with previous connectivity assessments by tract tracing in squirrel monkeys, cats, and rats (Gimenez-Amaya and Graybiel, 1990; Flaherty and Graybiel, 1993; Rajakumar et al., 1993).

### 3.6. Compartment-specific biases in striato-pallido-thalamic tractography

**Figure 2:**
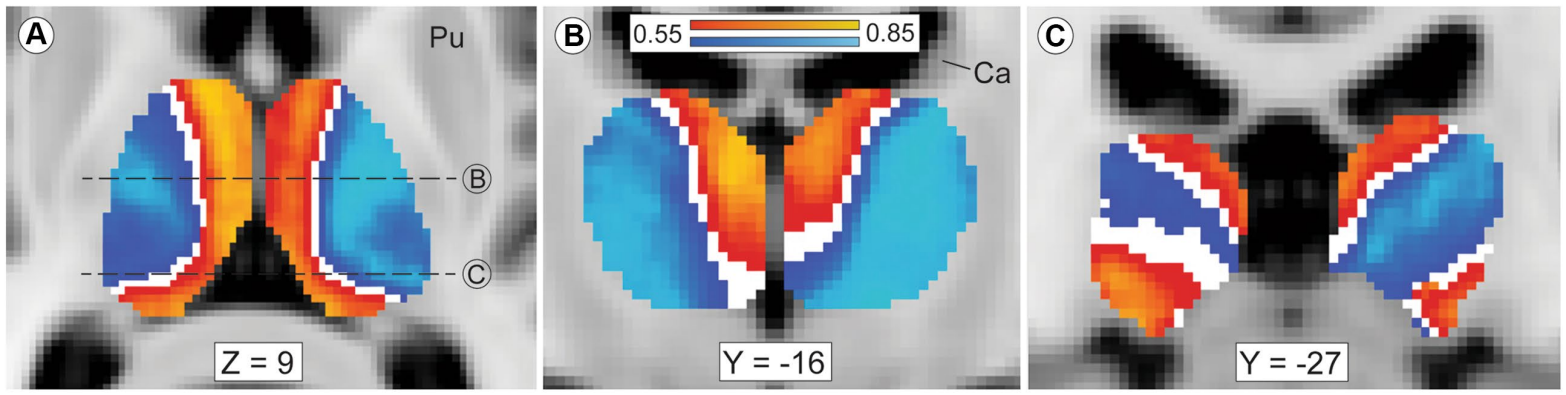
Mean probability of striato-pallido-thalamic connectivity for striosome-originating and matrix-originating streamlines. Compartment-specific biases in structural connectivity parallel the anatomic organization of the thalamus. In axial **(A)** and coronal **(B,C)** views, probability maps that favored striosome-like voxels (red-yellow) or matrix-like voxels (blue-light blue) were overlaid on the MNI_152_1mm template brain. Larger biases can be seen in yellow voxels (striosome-like) and light blue voxels (matrix-like). The coronal planes of section in B and C are indicated by dashed lines in A. Maps are the averaged probabilities from all subjects, with left and right hemispheres run independently. These views demonstrate probability values between 0.55–0.85, voxels with substantial compartment-specific bias in structural connectivity. Voxels with indeterminate bias (those with connection probabilities of 0.45–0.55) do not appear in either probability map (white voxels). The anterior and medial nuclear groups have strong bias towards striosome-like striatal voxels, while the lateral nuclear group is mostly biased towards matrix-like voxels. Voxels with indeterminate bias closely approximate the internal medullary lamina. The pulvinar nucleus, the caudal-most portion of the thalamus, has a divided pattern of connectivity, with some pulvinar zones biased towards matrix-like voxels and other zones biased towards striosome-like voxels (panel **C**). Note that in this mode of tractography (classification targets) each thalamic voxel was mapped individually – the similarity of these probability maps to prior histology-based divisions of thalamic nuclei underscores the fact that probabilistic tractography follows the intrinsic anatomic and functional anatomy of the thalamus. Images follow radiologic convention. Coordinates follow MNI convention. Ca, caudate; Pu, putamen.

Our thalamic CTT (Methods 2.5, tractography iteration three) measured striato-pallido-thalamic connectivity between the striatal compartments and each thalamic voxel. Though we assessed each thalamic voxel independently, compartment-specific biases in connectivity mirrored the large-scale anatomic organization of the thalamus (Figure 2). Similarly, while we performed CTT separately in left and right hemispheres, the patterns of structural connectivity in the two hemispheres were strikingly similar. Likewise, the mean connection probability for striosome-favoring and matrix-favoring thalamic voxels was highly similar in the left and right hemispheres (favoring matrix-like voxels: left, 0.72 SEM ± 0.024; right, 0.67 SEM ± 0.024 – favoring striosome-like voxels: left, 0.64 SEM ± 0.023; right, 0.67 SEM ± 0.022). Thalamic voxels whose connectivity was biased towards the matrix-like compartment were predominantly located within the lateral nuclear group. Thalamic voxels with bias towards the striosome- like compartment largely occupied the anterior, medial, and midline nuclear groups. Thalamic voxels with sub-threshold bias closely approximated the internal medullary lamina (white voxels in Figures 2A,B), though all measured intralaminar nuclei had biased connectivity. The pulvinar was unique in not conforming to this pairing of nuclear group and striato-thalamic connectivity (Figure 2C). Striosome-like and matrix-like connectivity cut across the pulvinar in orientations that did not match the pulvinar subdivisions we utilized (PuA, PuI, PuL, PuM). Note that while these qualitative patterns utilized the averaged probability maps (which included all subjects), our subsequent quantitative assessments of connectivity measured each nucleus in individual subjects.

**Figure 3:**
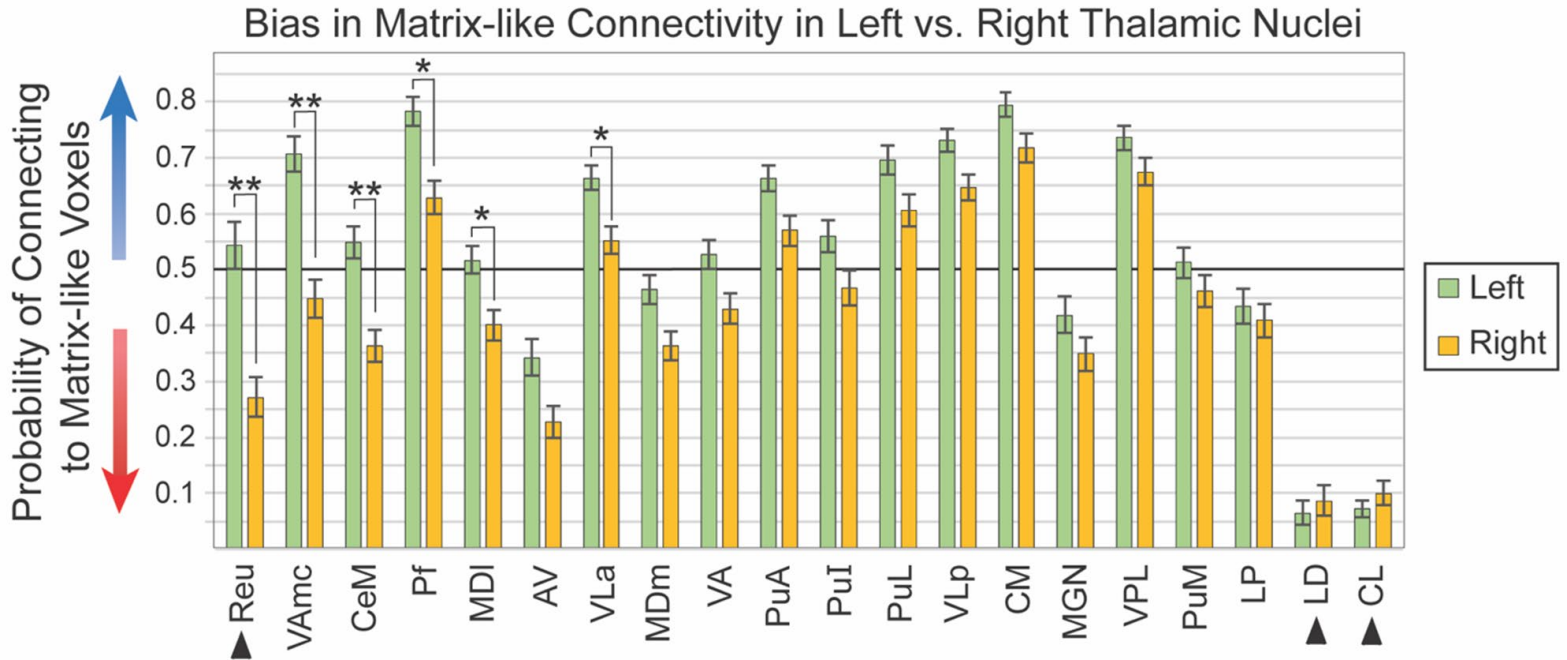
Connectivity to matrix-like voxels is stronger in the left hemisphere for most thalamic nuclei. Biased connectivity from the matrix-like compartment is larger in the left hemisphere. While this bias was significant in only six of 20 nuclei, bias was larger in the left for 18 of the 20 nuclei assessed. Note that the darker horizontal line (0.5) indicates the point of equal connectivity between thalamic seed voxels and matrix- or striosome-like target voxels. Higher values on the y axis indicate greater matrix-like connectivity (blue arrow), with lower values indicating greater striosome-like connectivity (red arrow). For any given nucleus, the values for matrix-like and striosome-like connectivity sum to one. Therefore, we present only the matrix-like connectivity for left and right hemispheres. Error bars indicate the standard error of the mean. Three nuclei whose data originated from the HCP cohort alone are indicated by arrowheads. Significance threshold: **, *p* < 2.5×10^−6^; *, *p* < 2.5×10^−3^.

Given the visual similarity of left and right hemispheres (Figure 2), we sought to identify whether thalamic nuclei in the left and right hemispheres shared the same patterns of striatal compartment connectivity. While hemispheric specialization in thalamic connectivity was interesting as a primary question, this assessment was also necessary to determine whether hemispheres could be combined for subsequent analyses or should be analyzed separately. We quantified striato-pallido-thalamic connectivity bilaterally for 20 thalamic nuclei (Figure 3, organized from maximum to minimum interhemispheric difference). For two nuclei (Pf, VLa), connectivity was significantly different between left and right hemispheres, but both were biased towards matrix-like voxels; the left hemisphere was simply more matrix-biased than the right (means - Pf: 0.78 vs. 0.63, *p* < 7.3×10^−5^; VLa: 0.66 vs. 0.55, *p* < 4.5×10^−4^). Note that striosome-like and matrix-like connectivity measures always summed to one. Therefore, we have presented only the measures of matrix-like connectivity. Four nuclei had significant differences in compartment-specific connectivity in one hemisphere, but neutral connectivity in the other hemisphere (presented as mean matrix-like connectivity in the left vs. right hemisphere - CeM: 0.55 vs. 0.36, *p* < 1.8×10^−6^; MDl: 0.52 vs. 0.40, *p* < 8.5×10^−4^; Reu: 0.54 vs. 0.04, *p* < 2.2×10^−6^; VAmc: 0.71 vs. 0.45, *p* < 1.4×10^−8^). Note that Reu values were derived only from the 100 subjects in our HCP cohort.

We performed post-hoc ANCOVA testing to identify demographic or experimental factors that contributed to interhemispheric differences in connectivity with the striatal compartments. We tested the following factors for all 20 thalamic nuclei: age, number of diffusion directions, scanner type, handedness, hemisphere, self-identified race, sex, and interactions between sex, race, diffusion directions, and scanner type. No demographic or experimental factor, and no interaction between factors, was a significant contributor to interhemispheric bias.

**Figure 4:**
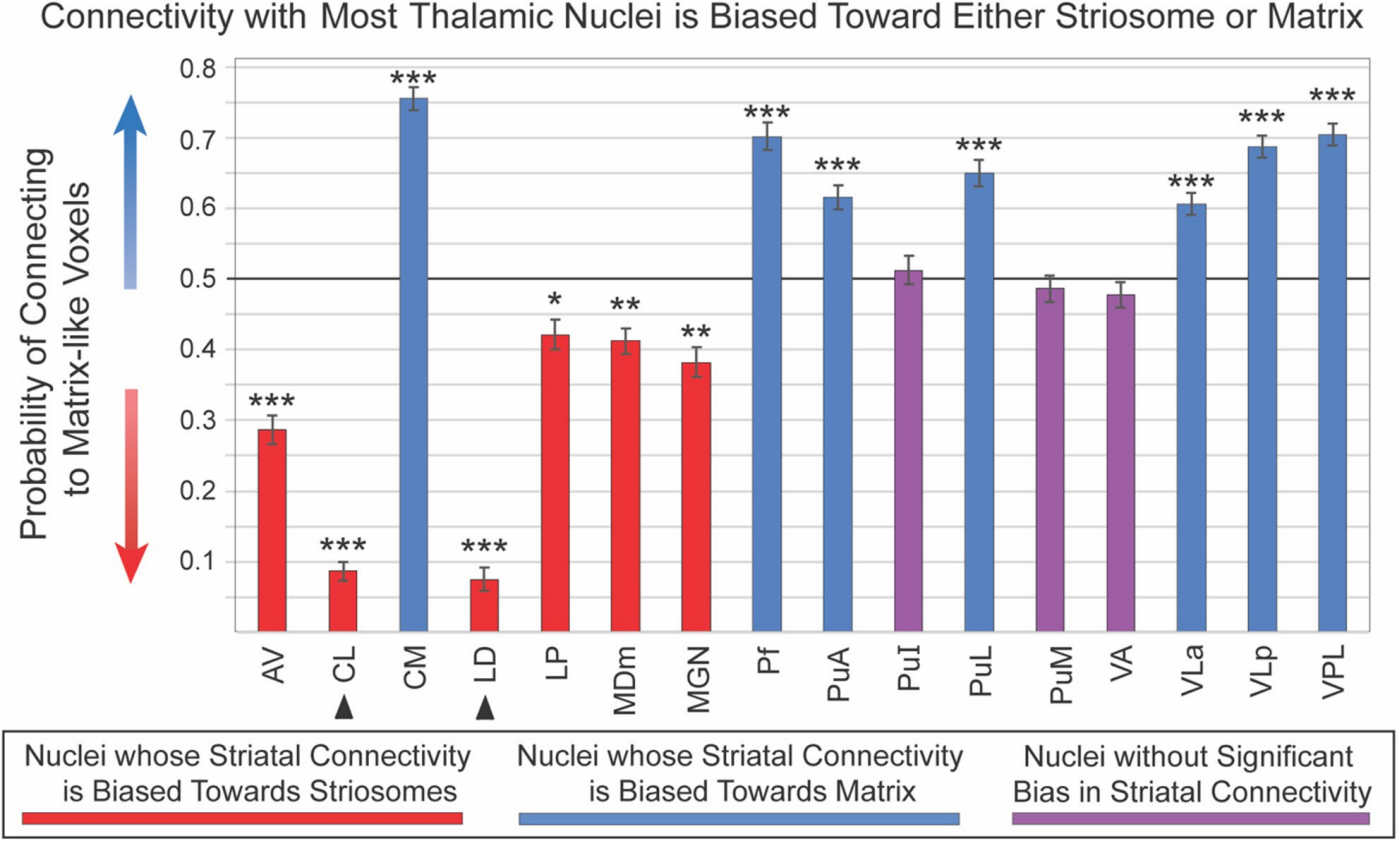
Connectivity with most thalamic nuclei is biased toward either striosome-like or matrix-like voxels. Disynaptic projections from striatum to thalamus (via the globus pallidus interna) are biased: streamlines that originate in striosome-like or matrix-like voxels largely reach different thalamic nuclei. Seven nuclei were dominated by projections from matrix-like voxels (blue bars), six nuclei were dominated by striosome-like projections (red bars), and three nuclei had no compartment bias (purple bars). Note that the darker horizontal line (0.5) indicates the point of neutral connectivity. Higher values on the y axis indicate greater matrix-like connectivity (blue arrow), with lower values indicating greater striosome-like connectivity (red arrow). For any given nucleus, the values for matrix-like and striosome-like connectivity sum to one. Therefore, we present only the matrix-like connectivity for each thalamic nucleus. Error bars indicate the standard error of the mean. Two nuclei whose data originated from the HCP cohort alone are indicated by arrowheads. Significance thresholds: *, *p* < 3.1×10^−3^; **, *p* < 3.1×10^−9^; ***, *p* < 3.1×10^−15^.

Sixteen thalamic nuclei did not differ significantly in their compartment bias between left and right hemispheres, and we therefore combined the hemispheres for subsequent analyses (Figure 4; Table 1): AV, CL, CM, LD, LP, MDm, MGN, Pf, PuA, PuI, PuL, PuM, VLa, VLp, and VLP. Note that CL and LD values were derived only from the 100 subjects in our HCP cohort. Six nuclei showed significant connectivity bias toward striosome-like voxels (mean connectivity of 0.5 indicated no bias, with 0 and 1 indicating complete bias towards striosome-like or matrix-like voxels, respectively): AV – 0.29, *p* < 9.6 × 10^−43^; CL – 0.09, *p* < 4.6 × 10^−155^; LD – 0.08, *p* < 2.1 × 10^−114^; LP – 0.42, *p* < 7.2 × 10^−8^; MDm – 0.41, *p* < 1.6 × 10^−11^; MGN – 0.38, *p* < 4.7 × 10^−14^. Seven regions showed significant connectivity bias towards matrix-like voxels: CM – 0.76, *p* < 2.0 × 10^−83^; Pf – 0.70, *p* < 1.0 × 10^−42^; PuA – 0.62, *p* < 7.2 × 10^−20^; PuL – 0.65, *p* < 3.8 × 10^−27^; VLa – 0.61, *p* < 4.6 × 10^−20^; VLp – 0.69, *p* < 2.4 × 10^−58^; VPL – 0.71, *p* < 1.7 × 10^−61^. The three remaining thalamic nuclei were not significantly biased toward either striatal compartment: PuI – 0.49, *p* < 0.37; PuM – 0.49, *p* < 0.31; VA – 0.48, *p* < 0.08. Upon visual assessment of individual subjects’ probability distributions, it was clear that the segmentations of PuI and PuM paralleled the division between striosome-favoring and matrix-favoring connectivity, but straddled the boundary between them. Assessment of these two pulvinar areas may therefore require more subtle thalamic nuclear segmentations.

**Table 1:** Probability of striato-pallido-thalamic connectivity with matrix-like voxels.

Nucleus	Mean	Standard Error	99.7% CI	*p* value
AV	0.29	0.021	[0.229, 0.345]	9.6×10^−43^
CL	0.09	0.013	[0.047, 0.125]	4.6×10^−155^
CM	0.76	0.017	[0.706, 0.806]	2.0×10^−83^
LD	0.08	0.017	[0.025, 0.125]	2.1×10^−114^
LP	0.42	0.020	[0.361, 0.482]	7.2×10^−8^
MDm	0.41	0.018	[0.358, 0.467]	1.6×10^−11^
MGN	0.38	0.022	[0.321, 0.444]	4.7×10^−14^
Pf	0.70	0.020	[0.645, 0.760]	1.0×10^−42^
PuA	0.62	0.018	[0.563, 0.669]	7.2×10^−20^
PuI	0.51	0.020	[0.454, 0.572]	0.37
PuL	0.65	0.019	[0.593, 0.707]	3.8×10^−27^
PuM	0.49	0.018	[0.431, 0.543]	0.31
VA	0.48	0.018	[0.422, 0.532]	0.077
VLa	0.61	0.016	[0.557, 0.656]	4.6×10^−20^
VLp	0.69	0.015	[0.642, 0.735]	2.4×10^−58^
VPL	0.71	0.016	[0.657, 0.753]	1.7×10^−61^

Structural connectivity with matrix-like voxels for the 16 thalamic voxels for which we combined left and right hemispheres. The confidence interval (CI) provided here (99.7%) matches the significance threshold utilized for this set of *t*-tests.

### 3.7. Mapping of striato-thalamic structural connectivity: human vs. model animals

Fourteen thalamic nuclei had (1) significant compartment-specific bias in striato-pallido-thalamic connectivity and (2) histology-based mapping in published animal studies with high-probability bias towards either striosome-like or matrix-like voxels (Table 2). In all 14 nuclei, our MRI-based method in humans matched the findings of histology-based studies in animals: AV, CL, CM, LD, MDl, MDm, MGN, Pf, PuA, PuL, Reu, VLa, VLp, VPL. We identified a bias toward striosome-like voxels in CeM, but prior animal studies were mixed, with some reporting a matrix bias (Berendse et al., 1988; Ragsdale and Graybiel, 1991) and others reporting a striosome bias (Prensa and Parent, 2001). Connectivity in the VAmc has not been mapped in animals, to the best of our knowledge. While thalamostriate connectivity was mapped for LP in rat, in an unfortunate mixing of terms between species, the rodent LP is the homologue of the pulvinar nucleus in primates (Funaki et al., 1998; Kamishina et al., 2008; Foik et al., 2020). Connectivity with the primate LP has not been described previously, to the best of our knowledge.

**Table 2:** Comparison of compartment-specific thalamic projections, animal and human assessments.

Thalamic nuclei	MRI-based results (striato-pallido-thalamic)	Animal Literature (thalamostriate and corticostriate)	Species utilized	Number of animals tested	Agree/Disagree/Unknown
CM	Matrix-biased	Matrix-biased	Cat^1^ Rat^2^ Squirrel Monkey^3^	29 93 4	Agree
Pf	Matrix-biased	Matrix-biased	Cat^1^ Squirrel Monkey^3^ Rat^2^	29 4 93	Agree
PuA	Matrix-biased	Matrix-biased	Tree Shrew^4^	9	Agree
VLa	Matrix-biased	Matrix-biased	Rat^5^ Cat^6^ Pig-Tailed Macaque^7^	36 6 5 (i)	Agree
VLp	Matrix-biased	Matrix-biased	Rat^5^ Cat^6^ Pig-Tailed Macaque^7^	36 6 5 (i)	Agree
VPL	Matrix-biased	Matrix-biased	Rat^5^ Cat^6^	36 6	Agree
PuL	Matrix-biased	Matrix-biased	Cat^6^ Tree Shrew^4^	6 9	Agree
AV	Striosome-biased	Striosome-biased	Cat^1^	29	Agree
CL	Striosome-biased	Striosome-biased	Cat^1^ Rat^2^ Rat^12^ Cat^6^	29 93 11 (i) 6	Agree
LD	Striosome-biased	Striosome-biased	Rat^8^	1	Agree
MDm	Striosome-biased	Striosome-biased	Cat^6^	6	Agree
MGN	Striosome-biased	Striosome-biased	Rat^9^	10 (i)	Agree
PuI	Neutral	Matrix-biased (central pulvinar)	Tree Shrew^4^	9	Unknown
PuM	Neutral	Matrix-biased (central pulvinar)	Tree Shrew^4^	9	Unknown
VA	Neutral	Matrix-biased Matrix-biased	Cat^6^ Cat^1^	9 29	Unknown
LP	Striosome-favoring	Unknown	None	None	Unknown
MDl	Striosome-biased	Striosome-biased	Monkey^10^ Rat^11^ Rat^2^	12 (i) 16 (i) 93	Agree
Reu	Striosome-biased	Striosome-biased	Rat^2^	93	Agree
CeM	Striosome-biased	Striosome-biased Matrix-biased	Rat^13^ Rat^2^	45 93	Mixed
VAmc	Matrix-biased	Unknown	None	None	Unknown

Comparison of our MRI-based findings in humans with histology-based assessments in animals. For 14 of 20 nuclei, our measures agree with prior animal literature. For the remaining six nuclei, bias is either mixed or unknown. For CeM, some tract tracing studies found a matrix-favoring bias and others found a striosome-favoring bias (“Mixed”). In LP and VAmc, we could identify no comparable study in animals (recorded as “Unknown” and “None”). In PuI, PuM, and VA, our method did not identify a compartment-specific bias (“Unknown”). We assessed the last four thalamic nuclei (blue) as separate hemispheres. The listed biases for these four nuclei are the mean of left and right hemispheres. Note that our MRI-based method utilized striatal voxels with striosome-like and matrix-like patterns of connectivity. While this inferential parcellation method replicates the compartment-specific spatial distribution, relative abundance, somatotopic organization, and connectivity profiles of striosome and matrix from animal and human histology, our use of living human subjects precluded the use of histologic confirmation of compartment identity. Superscripted numbers (“Species Utilized” column) correspond to the following citations, found in full form in the References Section: (1) Ragsdale and Graybiel (1991); (2) Unzai et al. (2017); (3) Sadikot et al. (1992); (4) Day-Brown et al. (2010); (5) Kincaid and Wilson (1996); (6) Avendano et al. (2006); (7) Pimenta et al. (2001); (8) Kamishina et al. (2008); (9) Barry et al. (2017); (10) Eblen and Graybiel (1995); (11) van Vulpen and Verwer (1989); (12) Wang and Pickel (1998); (13) Prensa and Parent (2001). The (i) designation (“Number of Animals” column) corresponds to the indirect association of compartment bias, as explained in the Discussion section.

## 4. Discussion

In this study we aimed to identify biases in striato-pallido-thalamic structural connectivity by assessing probabilistic tractography between parcellated striatal compartments (striosome-like and matrix-like voxels) and segmented thalamic nuclei. Prior studies in primate and non-primate species demonstrated that striosome and matrix have distinct functions, pharmacology, and extra-striate connectivity. Therefore, we propose that biases in compartment-specific structural connectivity may be a mode for regulating specific thalamic nuclei and cortico-striato-pallido-thalamo-cortical loops. If this same bifurcation in thalamostriate organization is present in humans, compartment-level regulation may be an anatomical basis for focused regulation of human motor and behavioral functions. We validated our compartment-specific biases in structural connectivity in humans (*in vivo*) with supporting anatomic findings (somatotopy and compartment bias) from previous animal studies, where available. Our findings concur with decades of tract tracing studies in animals and argue that in humans, striato-thalamic structural connectivity is biased towards either striosome-like or matrix-like voxels in most thalamic nuclei. Though it is encouraging that our MRI-based assessments were in agreement with prior animal and human histology and imaging studies, we remind readers that our striatal parcellations were inferential and based on differential connectivity – we did not distinguish striosome and matrix through immunohistochemical staining, the gold standard for identifying the striatal compartments.

It is important to consider the limitations of our tractography-based study. Probabilistic tractography is susceptible to false positive and negative streamline estimations, tract-specific biases based on orientation and degree of curvature, inter-individual variance in total streamline counts, and other potential confounds (Campbell and Pike, 2014). Given these risks, we validated our tractography results with animal and/or human histology, where available. Identifying voxels solely by their differential connectivity replicated the spatial distribution, abundance, extra-striate connectivity, and somatotopy demonstrated previously in animal and human tissue (Waugh et al., 2022). Further, we demonstrated that the precision of our striatal parcellations was essential for assessing compartment-like connectivity. Neighboring voxels (shifted by a mean of 1.8 voxels from their original, precise position) had either a complete loss or a marked reduction in compartment-specific connectivity. Finally, we refined our tractography to select for striato-pallido-thalamic connectivity by excluding streamlines that extended outside our subcortical bounding mask, and by imposing an obligatory waypoint (the GPi). We thereby reduced the contributions of false positive projections, such as thalamostriate projections and fibers of passage in the internal capsule. Since we included subjects from four distinct research cohorts, and measured voxel location at many sites in each subject, we assessed for the impact of repeated measures within each subject and the influence of experimental cohort. These factors did not influence intra-striate voxel location or compartment-specific bias. Finally, we quantified location and connectivity exclusively through within-subject comparisons to reduce the impact of inter-individual variance in streamline propagation. Our MRI-based measures of striato-pallido-thalamic connectivity in humans replicated and extended the findings of injected tract tracer studies in experimental animals. It is likely that streamlines we seeded within one thalamic nucleus propagated through other nuclei en route to our pallidal waypoint and striatal targets. Double-counts of a given streamline have the potential to inflate absolute measures of connectivity, and to inflate streamline counts in voxels closer to edges or the internal medullary lamina (as streamlines transit grey matter to reach white matter). However, it is not clear that double-counts would alter the bias in connectivity toward one striatal compartment. That is, two streamlines, seeded from the same thalamic voxel but bound for opposing striatal compartments, would progress through the same probability space in their egress from the thalamus, leading to double-counts for both compartments. As our method explicitly assesses bias in connectivity, not absolute numbers of streamlines, potential double-counts would not skew quantification toward one compartment. To avoid potential distortions by double-counting, we chose to quantify compartment-specific streamlines at each voxel, and always relative to the connectivity with the opposite striatal compartment. We observed no regional trends suggesting that strength or direction of connectivity bias was driven simply by proximity or location. Edge nuclei and embedded nuclei were equally likely to have compartment-specific biases in connectivity (Figures 2, 4). Two of our most-biased nuclei, CM and CL, are both intralaminar but were biased toward matrix-like and striosome-like voxels, respectively. While we cannot exclude the possibility that double-counts impacted our measures of compartment-specific bias, the agreement between our findings and prior tract tracing studies in multiple species (Table 2) argues that this potential limitation did not meaningfully alter our results.

Striatal parcellation – and thus, the entirety of our efforts to map striato-pallido-thalamic connectivity – depends on injected tract tracing studies in animals for both guidance and validation. It is therefore essential to consider the breadth and depth of these histologic studies when judging the veracity of our own findings. The strength of the evidence for compartment-specific connectivity varies considerably among the thalamic nuclei. Some thalamic nuclei were assessed in multiple studies, several species, and using multiple techniques to identify compartment-specific connectivity bias. Nuclei with the strongest evidence included CL, CM, Pf, PuA, PuL, VPL, LD, and AV. Other thalamic nuclei had limited numbers of investigations and generally mapped connectivity in only single species. These included the PuI, PuM, Reu, MDl, MDm, VL, and CeM. A few thalamic nuclei had never been mapped previously, to the best of our knowledge, or were mapped only inferentially by demonstrating thalamocortical connectivity with regions whose compartment-specific bias was demonstrated elsewhere. Nuclei with thin or absent evidence of compartment selectivity included the LP, MGN, VA, and VAmc. Further histologic mapping of striatothalamic, thalamostriate, and thalamocortical projections will be essential to validating our findings in these under-investigated thalamic nuclei. In an inversion of the typical relationship between animal and human studies, such MRI-based investigations in humans, with hundreds of subjects, may bolster the findings of histologic studies that included single or small numbers of animals.

An additional limitation for relating our results to prior histologic studies is the difficulty in comparing connectivity between techniques and across species. Our MRI-based measures of striato-pallido-thalamic connectivity are not a direct comparison to prior tracer-based techniques that assessed thalamostriate and corticostriate connectivity in animals. However, if the striatal limbs of CSTC loops are segregated through either striosome or matrix, we hypothesized that these compartment-specific biases would be shared among the multiple limbs of the CSTC loop (Unzai et al., 2017) – that striato-pallido-thalamic projections (MRI in humans) would match the biases of thalamostriate projections (injected tracers in animals).

Studies of compartment-level striatal projections to the globus pallidus in cats, rats, and squirrel monkeys showed that axons originating from matrix dominate striatopallidal connectivity (Gimenez-Amaya and Graybiel, 1990; Flaherty and Graybiel, 1993; Rajakumar et al., 1993). Our results replicated those findings: streamlines seeded from matrix-like voxels were 5.7-fold more likely to reach the GPi, despite the fact that striosome-like and matrix-like masks seeded equal numbers of streamlines. We previously demonstrated that streamlines seeded by striosome-like voxels contacted 16.1% *more* extra-striate voxels than streamlines seeded by matrix-like voxels (Waugh et al., 2022). Therefore, the dominance of streamlines seeded by matrix-like voxels in striato-pallidal projections is not due to a general reduction in connectivity of striosome-like voxels.

We found that streamlines from striosome- and matrix-like voxels occupied distinct parts of the GPi, with little overlap. This anatomic segregation was qualitatively symmetric between hemispheres, even though we produced left and right hemisphere tractography independently. Our findings align with the tractography-based findings of Bertino et al., who demonstrated that limbic cortices (striosome-favoring) selectively project to the rostral GPi, while sensorimotor cortices (matrix-favoring) selectively project to the caudo-medial GPi (Bertino et al., 2020). This compartment-specific somatotopy in humans matches that demonstrated in both primate and non-primate species. General topographic organization of striato-pallidal synaptic connectivity has been demonstrated in the rat (Sloot and Gramsbergen, 1994; Fujiyama et al., 2011), squirrel monkey (Johnson and Rosvold, 1971; Smith and Parent, 1986), and macaque (Saleem et al., 2002), with striosome and matrix projections partially overlapping but impinging on neurochemically distinct zones (Rajakumar et al., 1993, 1994; Stephenson-Jones et al., 2013). Similarly, in mice the afferents from striosome and matrix MSNs to the entopeduncular nucleus (EP, the rodent equivalent of the GPi) synapse on different subclasses of EP neuron that release different neurotransmitters, have different firing patterns, and project to different targets (Wallace et al., 2017). If humans share these intermixed but functionally distinct populations of GPi neurons, segregating projections from striosome and matrix MSNs to different somatotopic zones may provide the architecture for separable functional roles for the two compartments in humans. The similarity of our tractography-based findings to the histology-based findings in animals supports the validity of using these methods to map striatal compartment-level structural connectivity in human health and disease.

Thalamic nuclei are often assessed at the level of anatomic groups, most commonly as anterior, medial, midline, lateral, and intralaminar nuclei. Thalamostriate projections are often mapped within this schema, with multiple nuclei within a group mapped in parallel, such as all midline (Unzai et al., 2017), lateral (Avendano et al., 2006), or intralaminar nuclei (Ragsdale and Graybiel, 1991). The biases in striato-pallido-thalamic connectivity we demonstrated closely mirror that group-level organization, both qualitatively (Figure 2) and quantitatively (Figure 4). It is important to note, however, that the traditional nuclear groups are not uniform in their functions. Among the intralaminar group, animal and human histologic characterization demonstrated that rostral and caudal intralaminar nuclei have distinct patterns of connectivity, they are engaged in disparate functions, and they have different vulnerability to neurodegeneration (Henderson et al., 2000; Galvan and Smith, 2011; Cover and Mathur, 2021). In cats, the rostral intralaminar nuclei (especially CL and paracentral nuclei) preferentially innervate the striosome, while the caudal intralaminar nuclei (CM and Pf) primarily project to the matrix (Ragsdale and Graybiel, 1991; Fujiyama et al., 2019). Other authors found that the rostral intralaminar nuclei had a mixed pattern of connectivity (Fujiyama et al., 2019). Our measures of striato-pallido-thalamic connectivity in the intralaminar nuclei match these prior findings: CL (the sole rostral intralaminar nucleus large enough to measure here) was markedly biased towards striosome-like voxels, while CM and Pf strongly favored matrix-like connectivity. Our CTT-based parcellation of the thalamus – carried out at the level of individual voxels – recapitulates the organization of thalamic nuclei into nuclear groups. This emergent property suggests that striatal voxels identified by their differential connectivity are embedded within distinct striato-thalamic structural networks, and potentially, are embedded within distinct CSTC loops.

Thalamostriate projections originating in the CM, Pf, VPL, pulvinar, and ventrolateral (VL) favored the matrix compartment, or other regions highly biased toward the matrix compartment, in anterograde tracing studies in squirrel monkey, rat, cat, and tree shrew (Sadikot et al., 1992; Kincaid and Wilson, 1996; Avendano et al., 2006; Unzai et al., 2017). Our striato-pallido-thalamic connectivity data matches these matrix-biased thalamostriate findings. Tract tracing studies in the rat, cat, and squirrel monkey demonstrated that the VL projects primarily to matrix and receives projections from matrix-favoring portions of the EP (Rajakumar et al., 1993; Pimenta et al., 2001; Avendano et al., 2006). These VL tracing studies also support our findings of distinct, compartment-specific zones within the EP/GPi. Similarly, multiple types of histologic characterization in animals have demonstrated striosome-specific connectivity with particular thalamic nuclei. Anterograde tract tracing studies demonstrate that the medial and midline nuclei (such as Reu) generally project to striosome in animals (Goldman-Rakic and Porrino, 1985; Berendse et al., 1988; Ragsdale and Graybiel, 1991; Avendano et al., 2006; Unzai et al., 2017; Phillips et al., 2019). Injection of tracer in LD (rat) produced terminal labeling exclusively in the striosome compartment (Kamishina et al., 2008). In cats, radiolabeled amino acid injections in the rostral thalamic pole (primarily AV) elicited strong labelling of the lateral striosome (Ragsdale and Graybiel, 1991). In addition to direct evidence for compartment-specific projections, inferential evidence also supports compartment-specific thalamostriate projections. The CL has a robust projection to the dorsal striatum and has afferent and efferent connections with limbic cortices that also preferentially innervate the striosome compartment (Groenewegen, 1988; Eblen and Graybiel, 1995; Wang and Pickel, 1998). Similarly, the striatum receives convergent projections from the MGN and the auditory cortices (Chen et al., 2019). Electrical stimulation of the prelimbic and infralimbic cortices, areas shown to project primarily to striosome (Donoghue and Herkenham, 1986; Gerfen, 1989; Nisenbaum et al., 1998; McGregor et al., 2019), directly alters the neuronal activity of the MGN (Barry et al., 2017). Given the shared structural connectivity between these nuclei and cortical areas with strong striosome biases, these animal histology studies strongly suggest that CL and MGN project primarily to the striosome. The biases of streamlines seeded by striosome-like voxels that we demonstrated here agreed with these thalamostriate projections identified in animals. While the concurrence of our human and prior animal connectivity measures is reassuring, it is important to note that these findings are supportive of, but are not a direct test of our hypothesis. It may be possible to determine whether compartment-specific biases in striato-pallido-thalamic connectivity match the compartment selectivity of thalamostriate projections through colocalized injection of anterograde and retrograde tracers.

We identified one prior study that utilized multi-synaptic tracing to investigate striato-pallido-thalamic connectivity in rodents. Aoki et al. (2019) paired retrograde and anterograde tracing to demonstrate that limbic and motor cortices project through distinct striato-pedunculo-thalamo-cortical circuits. Likewise, they identified spatially distinct limbic and motor zones in the thalamus and striatum. However, they did not assess projections for compartment selectivity. Therefore, comparison of our findings to prior histology-based assessments in animals is limited to single-synapse tracing studies. While animal studies that analyzed single steps in the striato-pallido-thalamic projection (e.g., striatopallidal, or pallidothalamic) lend support to our compartment-specific findings, these isolated segments do not sum to a full tracing of compartment-specific striato-pallido-thalamic connectivity. In a parallel limitation, the probabilistic nature of tractography, coupled with the millimeter-scale resolution of diffusion imaging, ensures that our results will obscure less-abundant tracts and potentially merge tracts that are adjacent but distinct. Despite these limitations, our methods were sufficient to demonstrate that projections from striosome-like and matrix-like voxels occupy different paths to the GPi, occupy different volumes within the GPi, and have significant and substantial biases in their connectivity with thalamic nuclei that match the results of prior histologic assessments in animals. Future multi-synaptic tract tracing studies in animals will be necessary to validate our findings and more fully map the contributions of each striatal compartment to striato-pallido-thalamic arm of CSTC loops. While multi-synapse tracing poses an increased challenge relative to single-synapse tracing, this type of connectivity mapping has previously been accomplished for striato-pedunculo-thalamo-cortical circuits and even with projections from visceral organs to the brain (Bostan et al., 2010; Aoki et al., 2019; Dum et al., 2019; Foster et al., 2021).

The connectivity biases we localized in humans may not compare directly with findings in animals. For example, tract tracing studies did not subdivide the VL or pulvinar nuclei into subnuclei, as we did (Ragsdale and Graybiel, 1991). We found that projections to both VLa and VLp were highly biased toward matrix-like voxels, similar to the biases of the combined VL described in animals. However, in tree shrews the pulvinar was divided into two subdivisions, both of which projected to the matrix compartment (Day-Brown et al., 2010). In humans the pulvinar can be divided into four or more subdivisions. The anatomic correlation between human and tree shew pulvinar is therefore uncertain. In primates, the LP and pulvinar have distinct anatomic boundaries, peptide expression, and neurotransmitter profiles (Pérez-Santos et al., 2021), but these distinctions are not clearly established in non-primate species. Therefore, translating these posterior thalamic findings to human LP and pulvinar is problematic. For other thalamic nuclei, histologic mapping in animals is insufficient to identify a clear pattern of connectivity. Anterograde tracers injected into CeM predominantly reach the matrix (Berendse et al., 1988; Ragsdale and Graybiel, 1991). However, Prensa and Parent found that nigral neurons with dense projections to striosome also send collaterals to CeM (Prensa and Parent, 2001). We found no prior mapping of compartment-specific connectivity of the lateral mediodorsal (MDl), distinct from the other parts of the mediodorsal nucleus. However, the entire mediodorsal thalamus has prominent connectivity with cortical areas that selectively project to striosome, so MDl may also share striosome-favoring bias (Vogt et al., 1987; van Vulpen and Verwer, 1989; Eblen and Graybiel, 1995; O'Muircheartaigh et al., 2015; Phillips et al., 2019). Tract tracing results for Reu are limited but trend strongly toward striosome. In a single cat, a large injection of tracer that included both Reu and the ventromedial nucleus produced “marked” striosome labeling (Ragsdale and Graybiel, 1991). Reu is strongly interconnected with limbic structures (Vertes et al., 2022), which predominantly project to striosome (Donoghue and Herkenham, 1986; Gerfen, 1989; Graybiel, 1990). In contrast, mapping of a *single* Reu neuron identified a mixed, but matrix-dominated pattern of thalamostriate projections (Unzai et al., 2017). We could find no example of compartment-specific mapping of the VAmc. Greater characterization in animal models, especially primates, will be necessary to understand the thalamostriate and striatothalamic connectivity of the CeM, LP, MDl, pulvinar, Reu, and VAmc nuclei.

Each of the 20 thalamic nuclei we assessed had a particular bias in striatal connectivity: favoring striosome-like voxels, favoring matrix-like voxels, or neutral. However, for 18 out of 20 thalamic nuclei, the left hemisphere was more biased towards matrix than the right (Figure 2). This consistent bias was independent of the type of connectivity that dominated (i.e., present in both striosome-favoring and matrix-favoring nuclei), and was not influenced by handedness, type of diffusion protocol, or any demographic variable. While we executed tractography in each hemisphere independently, all experimental parameters (seed volume, number of streamlines seeded, waypoint and exclusion masks, etc.) were identical in left and right hemisphere tractography. Our hand-segmented thalamic masks were slightly larger on the left (6.6%), but as the targets of tractography (striosome-like and matrix-like striatal masks) were of equal volume, we cannot detect a reason this would skew connectivity from most thalamic nuclei toward matrix-like voxels. This asymmetry in thalamic volume likely reflects neuroanatomic reality – our findings are similar to those of Ahsan et al., who determined that the left thalamus in healthy adults was significantly larger than the right (Ahsan et al., 2007). This asymmetry in compartment bias was independent of a nucleus’ position within the thalamus (medial vs. lateral, rostral vs. ventral, embedded vs. edge). It is unclear how asymmetries in the edges of our left and right thalamic masks would produce such widespread, near-universal asymmetries in striatal connectivity. This left-sided bias towards matrix-like voxels was significant in 6 of 20 nuclei. Twelve additional nuclei were more biased in the left but not to a significant degree. The probability of 18/20 nuclei sharing a hemispheric bias by chance is 1 in 5,519. These structural asymmetries may also reflect laterality in function. Previous studies demonstrated that motor performance (speed, precision) was better in children whose functional connectivity was dominated by the left hemisphere (Barber et al., 2012). Likewise, increased motor control (manual dexterity) correlates with the degree of hemispheric lateralization for language (Hodgson et al., 2021). In contrast, abnormalities in lateralization may contribute to the movement disorder dystonia, in which both functional (Blood et al., 2004) and structural (Blood et al., 2019) asymmetries in motor control regions correlate with symptoms. Prior studies that utilized injected tract tracers to assess thalamic connectivity generally did so qualitatively. To fully address the possibility that the left thalamus is more biased toward matrix than the right, we suggest that future studies utilizing injected tract tracers in animals should *quantitatively* compare striatothalamic projections between the hemispheres.

Thalamic asymmetries have been identified in clinical neurology, in human brain mapping through neuroimaging and histology, and in animal neuroscience. The bias of the left thalamus towards the matrix-like compartment may be another example of these widespread thalamic asymmetries. Cerebral infarction, when limited to the thalamus, appears to have equal frequency in the left and right hemisphere. However, left thalamic infarctions are significantly more likely to produce clinically impactful symptoms (Rangus et al., 2022), leading left-sided thalamic infarction to be diagnosed at double the rate of right-sided thalamic infarction (Schaller-Paule et al., 2021). In healthy adolescents, thalamocortical structural connectivity was 24% greater in the left than the right thalamus (Alkonyi et al., 2011). Similarly, pallido-thalamic structural connectivity is significantly higher in the left thalamus, an asymmetry that is present in nuclei at every part of the rostro-caudal extent of the thalamus (Pelzer et al., 2017). During both innocuous and painful thermal stimulation, the right thalamus is significantly more activated than the left, regardless of which side of the body is stimulated (Coghill et al., 2001). Concentrations of the metabolite phosphocreatine, hypothesized to be a “substrate of wakefulness,” decline significantly from morning to afternoon, and are restored to morning levels by an afternoon nap – but this metabolic change occurs only in the left thalamus, not the right (Gordji-Nejad et al., 2018). Neurotransmitters are also lateralized in the human thalamus. The right thalamus has more μ-opioid receptor availability than the left, an asymmetry that is greater in the thalamus than in 15 other brain regions (Kantonen et al., 2020). Tissue norepinephrine is strongly lateralized in the postmortem human thalamus (Oke et al., 1978). In healthy rats, 98% of the brain’s mast cells are found in the thalamus. At most points in the rostro-caudal extent of the thalamus, the left thalamus has significantly more mast cells – up to 30% more (Goldschmidt et al., 1984). Asymmetries in mast cells number may have implications for human neuroinflammatory disorders, but as mast cell activation is also associated with improved goal-oriented behaviors and learning (Fitzpatrick and Morrow, 2017), asymmetries in humans, if present, may induce a lateralization in the motivational value of rewarding stimuli. These wide-ranging asymmetries in the anatomy, pharmacology, and metabolism of the thalamus provide context for the left-sided bias toward higher connectivity with matrix-like voxels, present in 18 of 20 thalamic nuclei. Our asymmetric findings may reflect a common feature of the mammalian thalamus.

Our method for parcellating the striatum into striosome-like and matrix-like compartments allowed us to map striato-pallido-thalamic connectivity *in vivo* in healthy humans. These techniques may also be useful for mapping striato-pallido-thalamic projections in developmental and degenerative disease states. Numerous disorders that were hypothesized to have a compartment-specific element to their pathology and progression may be more completely understood when studied in a compartment-specific approach, rather than at the whole-striatum level (Crittenden and Graybiel, 2011). For example, early in the clinical course of Huntington disease (HD) MSNs in the striosome degenerate more than those in matrix (Hedreen and Folstein, 1995; Matsushima, 2022). Striosome-dominated degeneration also holds true in later stages of HD for individuals whose symptoms were mood-predominant (Tippett et al., 2007). Selective degeneration of thalamic projections neurons has also been identified in HD, especially in the CM/Pf complex (Heinsen et al., 1996) and mediodorsal nucleus (Heinsen et al., 1999). These thalamic nuclei have opposing patterns of striatal compartment selectivity (CM/Pf, matrix-like; mediodorsal, striosome-like; Figure 4), suggesting that interpretating thalamic neurodegeneration in HD may require both symptom-specific (mood vs. motor) and compartment-specific assessments. Similarly, in both idiopathic Parkinson disease (PD) and progressive supranuclear palsy, thalamic neurodegeneration is relatively selective for the caudal intralaminar thalamic nuclei (CM, Pf; Henderson et al., 2000; Halliday, 2009). Though beyond the scope of this publication, we propose that denervation within compartment-specific CSTC loops may correlate with symptom type in PD as it appears to do for HD.

Abnormalities in the striatal compartments may be a feature of developmental disorders, in addition to the noted neurodegenerative disorders. For some developmental disorders, matrix- or striosome-specific neuropathologies are likely at the ultrastructural or interneuron level [e.g., schizophrenia (Roberts et al., 2005), Tourette syndrome (Kataoka et al., 2010)]. Such abnormalities are almost certainly below the resolution of diffusion MRI, a limitation of our striatal parcellation method. Other developmental disorders may have discernable structural abnormalities at MRI scale. Children with autism spectrum disorder were found to have increased ratios of matrix:striosome volume (identified by both immunohistochemical stains and mRNA expression, Kuo and Liu, 2020), an intriguing finding that will require repetition in larger cohorts. Habitual motor movements (stereotypies) have been hypothesized to arise from hyperactivation of striosomal neurons, based on studies in rats and squirrel monkeys (Canales and Graybiel, 2000; Saka et al., 2004). Stereotypies are common in autism and developmental disability (Goldman et al., 2009), but are also found among typically developing children (Singer, 2009; Oakley et al., 2015). Stereotypies are remarkably persistent throughout childhood (Tan et al., 1997; Valente et al., 2019) and frequently co-occur with a range of other childhood neurological disorders (e.g., motor developmental delay, attention-deficit hyperactivity disorder, obsessive compulsive disorder, Tourette syndrome), suggesting an anatomic susceptibility within this cohort of neurodevelopmental disorders (Tan et al., 1997; Valente et al., 2019). Whether these disorders are related to striosome or matrix dysfunction in humans has never been investigated, to the best of our knowledge. Anatomic relationships between each striatal compartment and particular symptoms can now be explored in living humans, and longitudinally through the course of diseases, for disorders associated with abnormal development, degeneration, or injury in specific CSTC loops. Identifying which thalamic nuclei have connectivity biases toward striosome-like or matrix-like voxels is an essential step in characterizing the role of the striatal compartments in specific CSTC loops, and thus in understanding the functions of striosome and matrix in human health and disease.

## Data availability statement

Publicly available datasets were analyzed in this study. This data can be found here: NIMH Data Archive, Data Object Identifier: 10.15154/1528201.

## Ethics statement

All research was conducted in accordance with the principles set forth in the Declaration of Helsinki. All data collection was approved by, and experimental oversight was conducted by, the Institutional Review Board for the respective institution where the subject was recruited. Oversight of the secondary analysis of MRI data was conducted by the Institutional Review Board of the University of Texas Southwestern. The participants provided their written informed consent to participate in the original studies that generated the MRI data presented here.

## Author contributions

AF: acquisition and analysis of data, original draft of the manuscript, and critical revising of the manuscript. AH, NB, NS, HB, and AB: acquisition of data and critical revising of the manuscript. JW: acquisition, analysis and interpretation of data, original draft of the manuscript, and critical revising of the manuscript. All authors contributed to the article and approved the submitted version.

## Glossary

**Table:** 

AP	Anterior to posterior
CSTC	Cortico striatal thalamo cortical
CTT	Classification targets tractography
DBS	Deep brain stimulation
DSC	Dice similarity coefficient
DTI	Diffusion tensor imaging
EP	Entopeduncular nucleus
FA	Fractional anisotropy
GPi	Globus pallidus interna
HCP	Human connectome project
HD	Huntington disease
MOR	Mu opioid receptor
MSNs	Medium spiny neurons
NDA	National Institute of Mental Health Data Archive
PA	Posterior to anterior
PD	Parkinson disease
ROI	Region of interest
SEM	Standard error of the mean
Thalamic nuclei were abbreviated in the convention of Iglesias et al. (2018):	
AV	Anteroventral
CeM	Central medial
CL	Central lateral
CM	Centromedian
LD	Laterodorsal
LP	Lateral posterior
MDl	Mediodorsal-lateral
MDm	Mediodorsal-medial
MGN	Medial geniculate nucleus
Pf	Parafascicular
PuA	Pulvinar-anterior
PuI	Pulvinar-inferior
PuL	Pulvinar-lateral
PuM	Pulvinar-medial
Reu	Reuniens-medial ventral
VA	Ventral anterior
VAmc	Ventral anterior magnocellular
VLa	Ventral lateral anterior
VLp	Ventral lateral posterior
VPL	Ventral posterolateral
