# Supplementary figures and images for: In humans, striato-pallido-thalamic projections are largely segregated by their origin in either the striosome-like or matrix-like compartments
Source: Front Neurosci. 2023 Oct 25;17:1178473. doi: 10.3389/fnins.2023.1178473 (PMC10634229; doi:10.3389/fnins.2023.1178473)

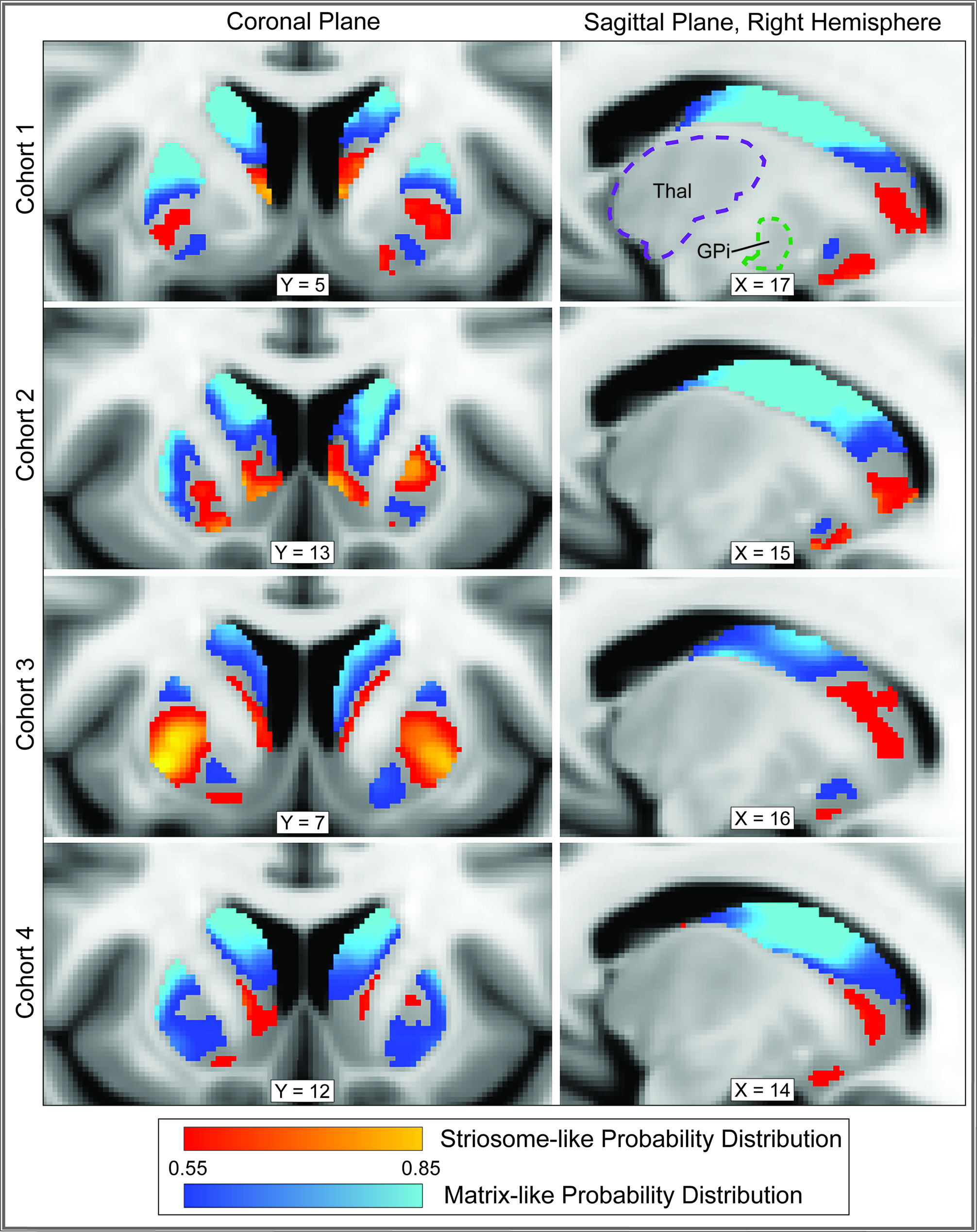

Supplement: Supplementary file 2 [file Figure_1.tif]

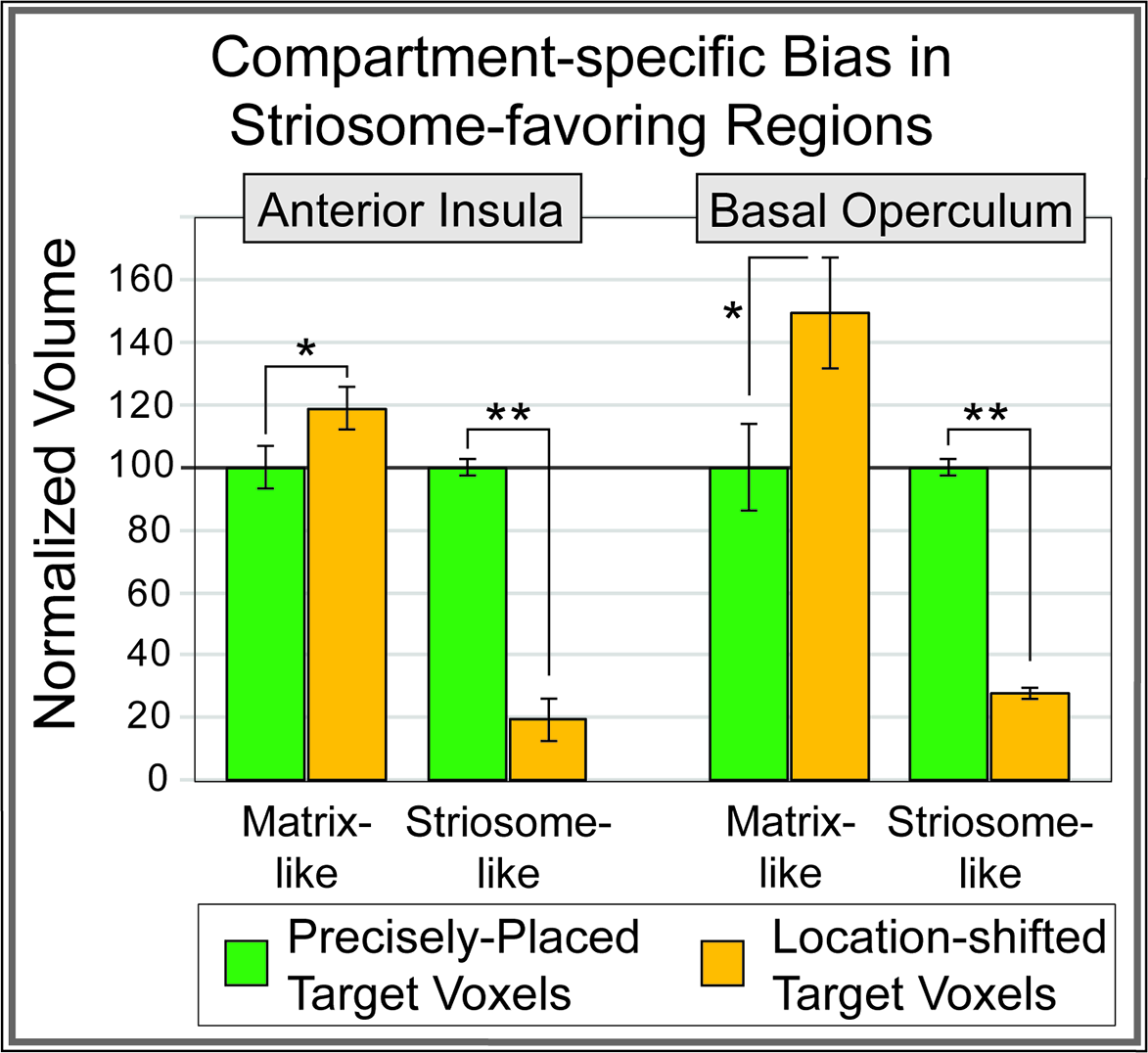

Supplement: Supplementary file 3 [file Figure_2.tif]
